# Cost-efficient multiplex PCR for routine genotyping of up to nine classical HLA loci in a single analytical run of multiple samples by next generation sequencing

**DOI:** 10.1186/s12864-015-1514-4

**Published:** 2015-04-18

**Authors:** Yuki Ozaki, Shingo Suzuki, Koichi Kashiwase, Atsuko Shigenari, Yuko Okudaira, Sayaka Ito, Anri Masuya, Fumihiro Azuma, Toshio Yabe, Satoko Morishima, Shigeki Mitsunaga, Masahiro Satake, Masao Ota, Yasuo Morishima, Jerzy K Kulski, Katsuyuki Saito, Hidetoshi Inoko, Takashi Shiina

**Affiliations:** Department of Molecular Life Science, Division of Basic Medical Science and Molecular Medicine, Tokai University School of Medicine, Isehara, Kanagawa 259-1143 Japan; HLA Laboratory, Japanese Red Cross Kanto-Koshinetsu Block Blood Center, Koto-ku, Tokyo 135-8639 Japan; Department of Hematology, Fujita Health University School of Medicine, Toyoake, Aichi 470-1192 Japan; Department of Legal Medicine, Shinshu University School of Medicine, Matsumoto, Nagano 390-8621 Japan; Division of Epidemiology and Prevention, Aichi Cancer Center Research Institute, Nagoya, Aichi 464-8681 Japan; Centre for Forensic Science, The University of Western Australia, Nedlands, WA 6008 Australia; Research Department, One Lambda Inc, Part of Thermo Fisher Scientific, Kittridge Street, Canoga Park, CA 91303-2801 USA

## Abstract

**Background:**

HLA genotyping by next generation sequencing (NGS) requires three basic steps, PCR, NGS, and allele assignment. Compared to the conventional methods, such as PCR-sequence specific oligonucleotide primers (SSOP) and -sequence based typing (SBT), PCR-NGS is extremely labor intensive and time consuming. In order to simplify and accelerate the NGS-based HLA genotyping method for multiple DNA samples, we developed and evaluated four multiplex PCR methods for genotyping up to nine classical HLA loci including HLA-A, HLA-B, HLA-C, HLA-DRB1/3/4/5, HLA-DQB1, and HLA-DPB1.

**Results:**

We developed multiplex PCR methods using newly and previously designed middle ranged PCR primer sets for genotyping different combinations of HLA loci, (1) HLA-DRB1/3/4/5, (2) HLA-DQB1 (3.8 kb to 5.3 kb), (3) HLA-A, HLA-B, HLA-C, and (4) HLA-DPB1 (4.6 kb to 7.2 kb). The primer sets were designed to genotype polymorphic exons to the field 3 level or 6-digit typing. When we evaluated the PCR method for genotyping all nine HLA loci (9LOCI) using 46 Japanese reference subjects who represented a distribution of more than 99.5% of the HLA alleles at each of the nine HLA loci, all of the 276 alleles genotyped, except for HLA-DRB3/4/5 alleles, were consistent with known alleles assigned by the conventional methods together with relevant locus balance and no excessive allelic imbalance. One multiplex PCR method (9LOCI) was able to provide precise genotyping data even when only 1 ng of genomic DNA was used for the PCR as a sample template.

**Conclusions:**

In this study, we have demonstrated that the multiplex PCR approach for NGS-based HLA genotyping could serve as an alternative routine HLA genotyping method, possibly replacing the conventional methods by providing an accelerated yet robust amplification step. The method also could provide significant merits for clinical applications with its ability to amplify lower quantity of samples and the cost-saving factors.

**Electronic supplementary material:**

The online version of this article (doi:10.1186/s12864-015-1514-4) contains supplementary material, which is available to authorized users.

## Background

The Human Leukocyte Antigen (HLA) or the Major Histocompatibility Complex (MHC) is a highly polymorphic region of the human genome (on the short arm of chromosome 6) that is critically involved in the rejection and graft-versus-host disease (GVHD) of hematopoietic stem cell transplants [1,2], the pathogenesis of numerous autoimmune diseases [3-6], infectious diseases [7] and drug adverse reactions [8,9]. Many variations of the conventional HLA genotyping methods such as incorporating restriction fragment polymorphisms (RFLP) [10], single strand conformation polymorphism (SSCP) [11], sequence specific oligonucleotides (SSOs) [12], sequence specific primers (SSPs) [13] and sequence based typing (SBT), like the Sanger method [14], have been used for the efficient and rapid HLA matching in transplantation therapy [15-18], research into autoimmunity and HLA related diseases [19,20], population diversity studies [21-23] and in forensic and paternity testing [24]. The HLA genotyping methods mainly applied today are PCR-SSOP, such as the Luminex commercial methodology [25,26], and SBT by the Sanger method employing capillary sequencing based on chain-termination reactions [14,27]. However, both methods often detect more than one pair of unresolved HLA alleles because of chromosomal phase (*cis/trans*) ambiguity [28-30]. To solve the phase ambiguity problem, we previously reported the development and application of the super high resolution-single molecule-sequence-based typing (SS-SBT) method using long-range PCR of the sample DNA from the promoter-enhancer region to the 3′ untranslated region (3′UTR) for 11 classical HLA loci, HLA-A, HLA-B, HLA-C, HLA-DRB1, HLA-DRB3/4/5, HLA-DQA1, HLA-DQB1, HLA-DPA1, and HLA-DPB1 in combination with next generation sequencing (NGS) platforms such as Ion PGM (Life Technologies) and GS Junior (Roche) [31-33]. Other long-range PCR and NGS-based HLA genotyping methods using 454 GS-FLX (Roche) and MiSeq (Illumina) platforms [30,34-36] also resolved the phase ambiguities. Thus, the NGS methods combined with the long-range PCR technology are expected to produce genotyping results to the field 4 level (formerly known as 8-digit typing) allelic resolution to efficiently detect new and null alleles without phase ambiguity.

**Figure 1:**
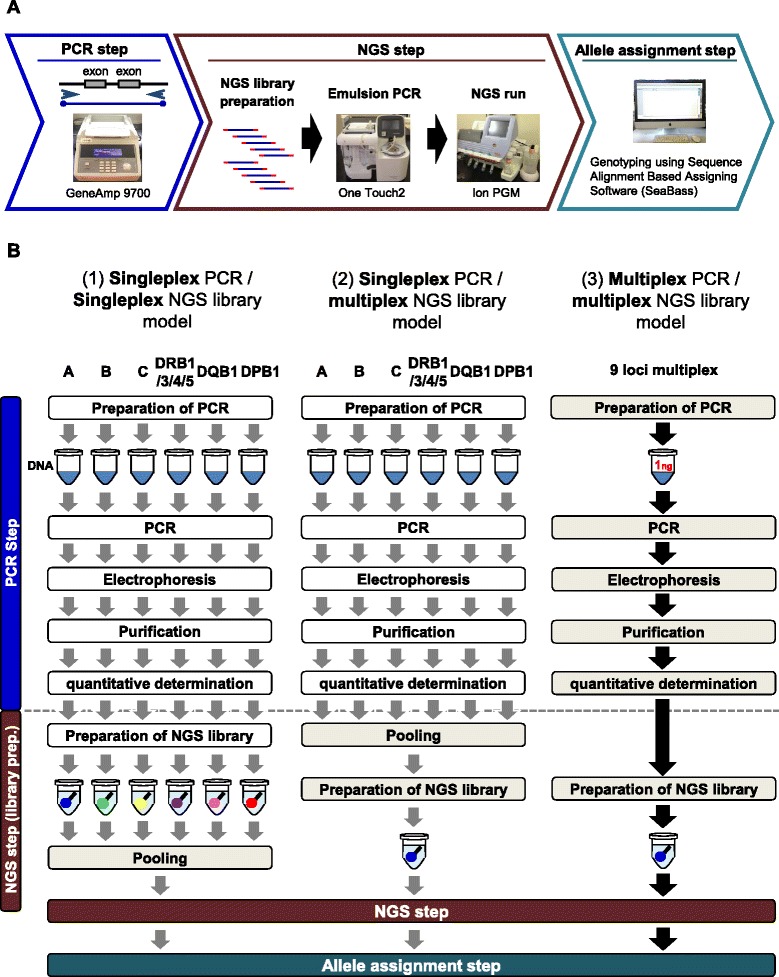
Workflow by the Ion PGM sequencer **(A)** and three different nine loci HLA genotyping procedures by PCR and NGS **(B)**. White and gray boxes indicate experimental steps using either several micro-tubes or a single micro-tube, respectively. Numbers in micro-tubes indicate template DNA amounts (ng).

The NGS methods are usually divided into three basic steps, long-range PCR of the DNA samples, NGS, and allele assignment step (Figure 1A) [25]. Before performing the NGS step there are at least five sub-steps for PCR, such as preparation of DNA template and PCR mixes, the PCR runs, electrophoresis, purification, and quantitative determination of the PCR products (Figure 1B). Multiple micro-tubes are required for the singleplex PCR. For example, at least six micro-tubes are required to amplify nine loci per DNA sample (Figure 1B). At least two kinds of NGS library preparation processes can be used after performing the long-range singleplex PCR procedure. One process is to prepare a number of single locus tagging NGS libraries and then pool all of them into a single NGS library (singleplex PCR/singleplex NGS library model: Figure 1B (1)). The other process is to pool all of the PCR products and prepare a single NGS library for each of the tagged multiple loci as a multiplex NGS library (singleplex PCR/multiplex NGS library model: Figure 1B (2)). However, the long-range singleplex PCR amplification and NGS library preparation as outlined in Figures 1B (1) and B (2) are extremely labor intensive and time consuming. Furthermore, it is easy to make human errors at the pooling stage that negatively influence the sequence read numbers. Therefore, simplification, acceleration and cost-saving in the NGS protocols are required if they are to become routine DNA typing methods and replace the conventional HLA genotyping methods such as SBT and PCR-SSOP (e.g. Luminex methodology).

In this paper, we describe the development and evaluation of four types of multiplex PCR methods that genotype multiple HLA loci to the field 3 level (6-digit typing) using combinations of locus specific PCR primers for up to nine classical HLA loci (HLA-A, HLA-B, HLA-C, HLA-DRB1/3/4/5, HLA-DQB1, and HLA-DPB1). We evaluated the uniformity and accuracy of NGS-based HLA genotyping among the nine HLA loci and between HLA alleles obtained by one of the multiplex PCR methods (the nine loci [9LOCI] multiplex method) in a single NGS run with the Ion PGM sequencer using 46 genomic DNA reference samples from Japanese subjects who represented a distribution of more than 99.5% HLA alleles in each of the HLA locus in the Japanese population. In addition, we investigated template DNA amounts as low as 1 ng to evaluate the smallest amounts of genomic DNA samples that we could use successfully in our multiplex PCR methods for NGS-based HLA genotyping.

## Results

### Characteristics of four types of multiplex PCR methods

**Figure 2:**
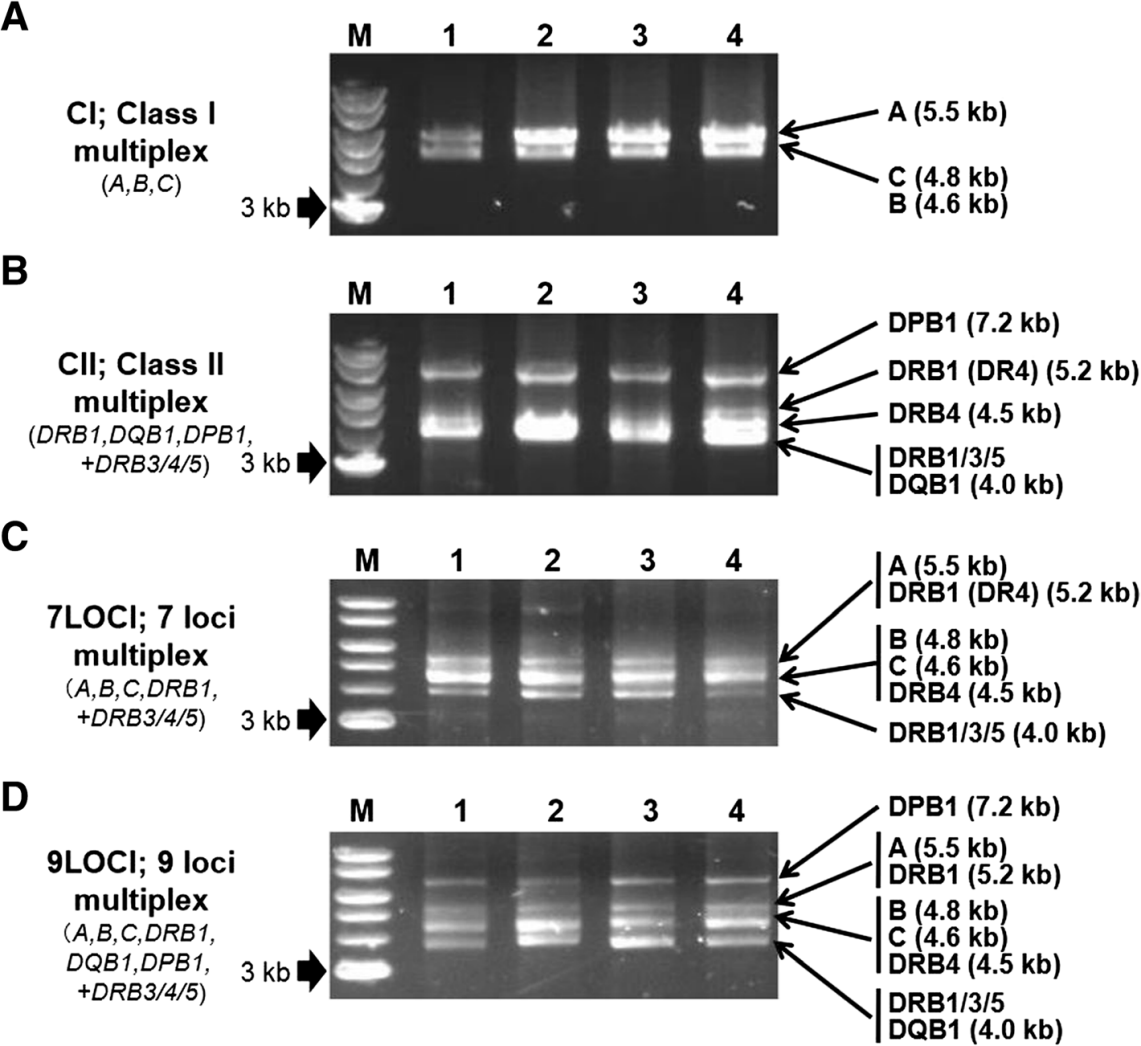
Band patterns of the multiplex PCR products. Electrophoresis images of PCR products from four unrelated genomic DNA samples (labeled 1 to 4) using four types of multiplex PCR methods, CI **(A)**, CII **(B)**, 7LOCI **(C)** and 9 LOCI **(D)**, respectively. Numbers 1 to 4 above the lanes indicate genomic DNA samples WW035, WW090, WW102, and WW104, respectively. The lanes labeled M are bands of the 1 kb DNA size marker ladder. The size of the bands and the HLA loci amplified by PCR are indicated on the right side of the figure.

Four types of multiplex PCR methods were developed after optimization of primer composition and PCR conditions such as annealing and extension temperatures using the HLA-A, HLA-B, HLA-C, HLA-DRB1/3/4/5, HLA-DQB1, and HLA-DPB1 specific primer pair sets (Figure 2). These four types were CI: A/B/C, CII: DRB1/3/4/5/DQB1/DPB1, 7LOCI: A/B/C/DRB1/3/4/5, and 9LOCI: A/B/C/DRB1/3/4/5/DQB1/DPB1. Two, three, three, and four bands that reflect the targeted PCR products were observed in CI, CII, 7LOCI, and 9LOCI multiplex PCR methods, respectively. Although most of the bands overlapped because of their similar PCR lengths as for HLA-B and HLA-C in CI (Figure 2A), it is noteworthy that HLA-A in CI (Figure 2A), and HLA-DPB1 in CII and 9LOCI (Figure 2B, D) were clearly observed as unique bands. In addition, the PCR product from the HLA-DRB1 gene varies in size depending on the DR sub-type such as 5.2 kb in the DR4 sub-type and 4.0-4.1 kb in the other DR sub-types.

When we applied the nine loci multiplex PCR (9LOCI) method using 46 genomic DNA samples (JPN01 to JPN46), the PCR products with similar band patterns were observed in all samples, although weak bands were also observed in some samples such as JPN23, JPN24, JPN25, and JPN29 (Additional file 1: Figure S1).

### Sequence read information obtained from 46 genomic DNA samples

Sequence read information was obtained for all the 46 genomic DNA samples after sequencing of the 9LOCI products using the Ion PGM system in a single sequencing run after gathering the 46 barcode-labeled DNA libraries into one tube. Draft read numbers in total were 5,284,570 sequence reads with a range of reads from 83,680 in JPN21 to 156,157 sequence reads in JPN45 (114,882 ± 20,482 standard deviation (SD) on average) that were high quality sequence reads with more than 10 quality values (QV) and an average QV of 28.0 ± 0.2 in the high quality sequence reads. The draft read bases in total were 1,447 Mb with a range between 21.7 Mb in JPN021 and 43.4 Mb in JPN041 (31.4 ± 6.0 Mb on average), with an overall average read length of 273.3 ± 9.9 bases and an overall mode read length of 358.6 ± 16.7 bases (Additional file 2: Table S1). Therefore, the sequence reads had high quality and sufficient sequence volume for further HLA genotyping analysis.

### Genotyping to the field 3 level on the nine HLA loci

Nucleotide similarity searches of the sequenced HLA alleles to the field 3 level using the BLAT program identified 276 alleles at the six HLA loci (Additional file 3: Table S2), except for DRB3/4/5, that were consistent with known HLA alleles assigned by the conventional Luminex method (Additional file 4: Table S3). Of the heterozygous 242 alleles, 224 were defined as two separate HLA alleles without any ambiguities. However, one locus observed in JPN15 (DPB1*05:01:01/DPB1*135:01 and DPB1*25:01) was not defined fully due to the absence of an informative SNP in exon 4 for DPB1*25:01 that was needed to assign the correct allele at the locus. Also, five types of ambiguous HLA alleles, such as DRB1*04:07:01/*04:92, DRB1*04:10:01/*04:10:03, DRB1*09:01:02/*09:21, DRB1*12:01:01/*12:10, and DPB1*13:01/*107:01 at a total of 17 loci, were observed because the informative SNPs that differentiate between ambiguous alleles were located outside of the PCR regions such as within exon 1 or exon 4 of HLA-DRB1 or HLA-DPB1 (Additional file 3: Table S2).

From the results of genotyping to the field 3 level, five HLA-DRB3, three HLA-DRB4, and three HLA-DRB5 alleles were assigned in the 45 DNA samples (Table 1 and Additional file 3: Table S2). There were thirty DRB1-DRB3/4/5 haplotypes in total with 15 assigned as the DRB1-DRB3 haplotype, 12 as DRB1-DRB4, and three as DRB1-DRB5. These haplotypes were identified by estimating HLA-DRB1 and HLA-DRB3/4/5 alleles without any descrepancy to previously reported DRB structures [32,37].

**Table 1:** **DRB1-DRB3/4/5 haplotypes to the field 3 level**

**DR haplotype**	**DR type**	**DRB1 type**	**DRB1 - DRB3/4/5 haplotype**		**Observed number**
			**DRB1 allele**	**DRB3/4/5 allele**	
DR52	DR3	DR3	DRB1*03:01:01	DRB3*02:02:01	1
	DR5	DR11	DRB1*11:01:01	DRB3*02:02:01	3
			DRB1*11:19:01	DRB3*02:02:01	1
		DR12	DRB1*12:01:01/*12:10	DRB3*01:01:02	4
			DRB1*12:01:01/*12:10	DRB3*01:12	1
			DRB1*12:02:01	DRB3*03:01:03	3
	DR6	DR13	DRB1*13:01:01	DRB3*01:01:02	1
			DRB1*13:02:01	DRB3*03:01:01	2
			DRB1*13:07:01	DRB3*02:02:01	1
		DR14	DRB1*14:02:01	DRB3*02:02:01	1
			DRB1*14:03:01	DRB3*01:01:02	2
			DRB1*14:05:01	DRB3*02:02:01	1
			DRB1*14:06:01	DRB3*02:02:01	1
			DRB1*14:07:01	DRB3*02:02:01	1
			DRB1*14:54:01	DRB3*02:02:01	1
DR53	DR4	DR4	DRB1*04:01:01	DRB4*01:02	1
			DRB1*04:03:01	DRB4*01:03:01	1
			DRB1*04:04:01	DRB4*01:03:01	1
			DRB1*04:05:01	DRB4*01:03:01	8
			DRB1*04:05:01	DRB4*01:03:02	2
			DRB1*04:06:01	DRB4*01:03:01	6
			DRB1*04:06:01	DRB4*01:03:02	1
			DRB1*04:07:01/*04:92	DRB4*01:03:02	1
			DRB1*04:10:01/*04:10:03	DRB4*01:03:01	1
	DR7	DR7	DRB1*07:01:01	DRB4*01:03:01	2
	DR9	DR9	DRB1*09:01:02/*09:21	DRB4*01:03:02	10
			DRB1*09:01:02/*09:21	DRB4*01:03:01	2
DR51	DR2	DR15	DRB1*15:01:01	DRB5*01:01:01	4
		DR15	DRB1*15:02:01	DRB5*01:02	5
		DR16	DRB1*16:02:01	DRB5*02:02	1

*Bold letter indicates allele assigned by published long-range PCR system [31].

Moreover, mapping analysis including other exons and introns using the SEABASS program suggested that no recombinations were evident within the gene loci examined for the 46 genomic samples (data not shown). Through this process one synonymous substitution was newly detected in exon 4 of HLA-C*07:04 of JPN16.

### Evaluation of the 9LOCI multiplex PCR method

**Figure 3:**
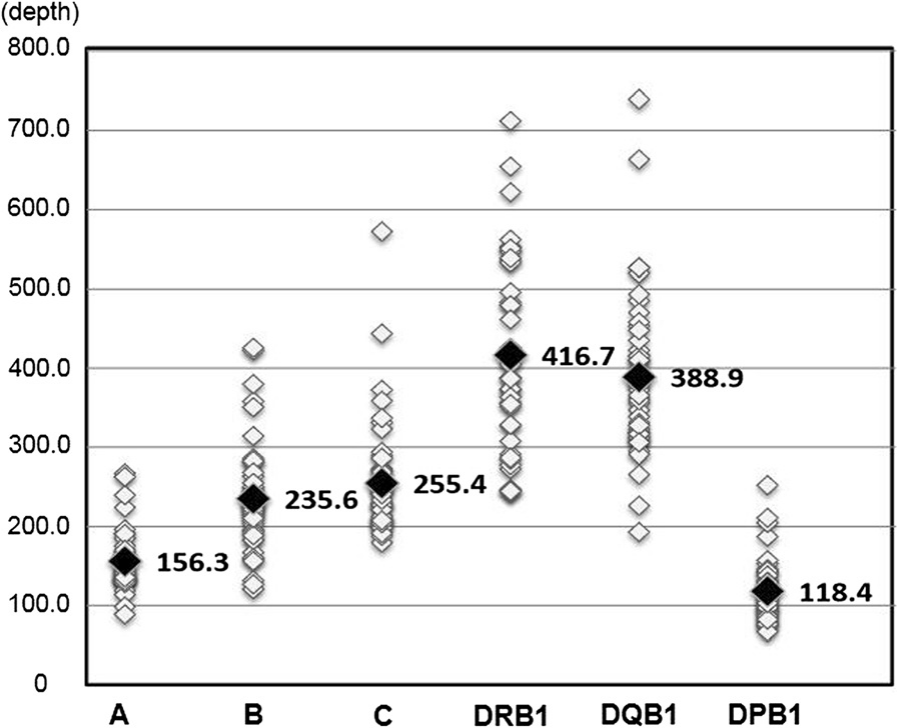
Locus balance depth plot based on normalized average depth per allele. White and black dots indicate normalized depth per locus in each DNA sample and normalized depth on average, respectively. The Y-axis of the plot shows the units of sequence depth and the X-axis shows the HLA gene loci. The detailed information is provided in Additional file 5: Table S4.

To evaluate the 9LOCI method, we compared the depth of redundancy derived from the sequence read numbers between HLA alleles and among HLA loci. An observed average depth and range for six HLA loci was as follows: 78.5 ± 42.0 from 31.6 to 225.1 for HLA-A, 116.5 ± 51.1 from 33.7 to 341.8 for HLA-B,130.0 ± 59.3 from 62 to 331.3 for HLA-C, 209.1 ± 115.9 from 44.1 to 712.4 for HLA-DRB1, 194.7 ± 104.9 from 74.9 to 614.4 for HLA-DQB1, and 59.2 ± 34.6 from 25.3 to 161.4 for HLA-DPB1 (Table 2 and Additional file 5: Table S4). The average depth ratio was mostly even for both alleles, ranging from 0.9 ± 0.3 in HLA-DRB1 to 1.0 ± 0.1 in HLA-A, but allelic imbalances of 0.2-0.5 were observed in eight DNA samples of HLA-B and 16 DNA samples of HLA-DRB1 (Additional file 5: Table S4). Most of the loci contained specific HLA allele groups such as B*39 and DRB1*04. On the other hand, an observed average depth among the HLA locus was from 116.3 ± 42.6 in HLA-DPB1 to 418.3 ± 143.0 in HLA-DRB1. When we normalized the values using the average sequence read numbers (114,882 reads), the depth was from 118.4 ± 37.8 in HLA-DPB1 to 416.7 ± 114.3 in HLA-DRB1 (Table 2). A locus balance plot showed locus imbalance among the loci ranging from high at HLA-DRB1 and HLA-DQB1 to low at HLA-A and HLA-DPB1 (Figure 3). However, the genotypes obtained at all the loci (276 alleles) in this study, were consistent with known HLA alleles to the field 3 level with more than 25 depth units per allele in DPB1*05:01:01 of JPN39, suggesting that the locus balance completely made up for the allelic imbalances observed for some specific alleles. Taken together, the 9LOCI PCR and NGS is a precise HLA genotyping method with relevant locus balance and without excessive allelic imbalance (<0.2) affecting the results deleteriously.

**Table 2:** **Depth information of each allele and each locus**

**Locus**	**Average depth (average ± SD)**		Average depth ratio (average ± SD)*	**Average depth per locus (average ± SD)**	
	**Observed**	Normalized*		**Observed**	Normalized*
HLA-A	78.5 ± 42.0	78.2 ± 37.9	1.0 ± 0.1	156.9 ± 48.5	156.3 ± 36.2
HLA-B	116.5 ± 51.1	117.8 ± 46.8	0.9 ± 0.2	233.0 ± 75.2	235.6 ± 68.2
HLA-C	130.0 ± 59.3	127.4 ± 55.8	0.9 ± 0.1	257.1 ± 90.9	255.4 ± 71.3
HLA-DRB1	209.1 ± 115.9	208.4 ± 108.2	0.9 ± 0.3	418.3 ± 143.0	416.7 ± 114.3
HLA-DQB1	194.7 ± 104.9	194.4 ± 95.3	0.9 ± 0.1	389.3 ± 115.9	388.9 ± 100.4
HLA-DPB1	59.2 ± 34.6	59.2 ± 31.3	0.9 ± 0.1	116.3 ± 42.6	118.4 ± 37.8

*calculated using the average draft read number (114,882 reads) shown in Additional file 2: Table S1.

### Investigation of template DNA amounts for the 9LOCI method

**Figure 4:**
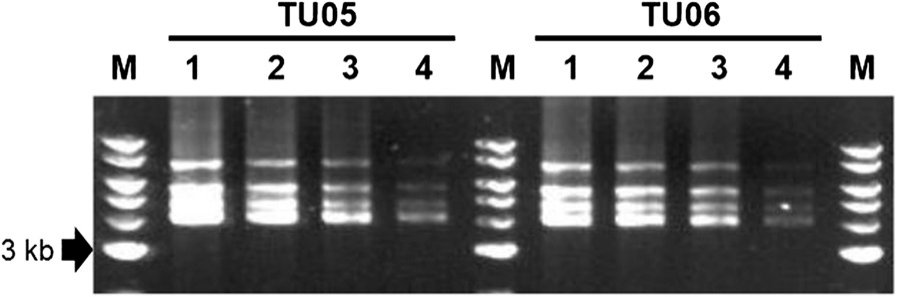
Electrophoresis images of PCR products from two unrelated genomic DNA samples TU05 and TU06 using the 9LOCI method. Numbers 1 to 4 above the lanes indicate the template DNA amounts 25 ng, 10 ng, 5 ng, and 1 ng, respectively. The lanes labeled M are bands of the 1 kb DNA size marker ladder.

In order to achieve precise HLA genotyping for the 9LOCI method even with using extremely small amounts of genomic DNA samples, we tried the 9LOCI multiplex PCR using four different amounts of template DNA, 1 ng, 5 ng, and 10 ng, along with standard amount of 25 ng. Amplified PCR products were observed for all of the template DNA amounts ranging from 1 to 25 ng (Figure 4). The DNA amounts after purification of the PCR products ranged from 119.7 ng (1 ng template) to 608.6 ng (25 ng template) in TU5, and from 112.5 ng (1 ng template) to 559.7 ng (25 ng template) in TU6. The purified PCR products of 100 ng were used for construction of Ion PGM libraries. The molarities after construction of Ion PGM libraries ranged from 8,444 pM (5 ng template in TU5) to 26,772 pM (25 ng template in TU5), and the sequence read numbers ranged from 329,752 (1 ng template in TU5) to 651,450 (25 ng template in TU6). The genotype results obtained for the eight samples used in this test of template DNA amounts were consistent to the previously assigned HLA alleles [31].

## Discussion

In this study, we used a reference set of 46 Japanese subjects that represented a distribution of more than 99.5% of the Japanese HLA alleles at the nine HLA loci genotyped by the multiplex PCR-NGS method using the Ion PGM system. All of the genotypes and linkages of DRB1 and DRB3/4/5 alleles were consistent with known alleles (Additional file 3: Table S2) and previous publications [32,37], suggesting that a combination of our multiplex PCR methods and the Ion PGM system is an efficient and accurate HLA genotyping method for the detection of HLA alleles to the field 3 level of genotyping without phase ambiguity. In addition, PCR products were obtained from all of the HLA loci by the multiplex PCR-NGS methods in tests using 400 non-Japanese (mainly European), subjects, confirming that the methods will be useful for the Japanese as well as for other world-wide populations (data not shown).

The high density in the average depth of sequences by NGS suggests that an increase in the DNA sample numbers for sequencing beyond 46 per run as described here is likely to contribute to even lower costs. For example, 85,879 sequence reads for sample JPN33 that was imbalanced at DRB1*04:06:01 was assigned with a sequence depth of 58.9. When we assume that an average of 85,879 sequence reads was obtained from a total of 5,284,570 reads that have a similar quality to those described in Additional file 2: Table S1, then at least 61 DNA samples could have been genotyped in a single run using Ion PGM. The multiplex PCR methods for HLA genotyping could also be used on other NGS platforms such as MiSeq (illumina), GS Junior and 454 GS-FLX (Roche), as well as on the 3^rd^ generation sequencing platform PacBio RS (Pacific Bioscience) that is based on single molecule real-time (SMRT) technology (unpublished data).

**Figure 5:**
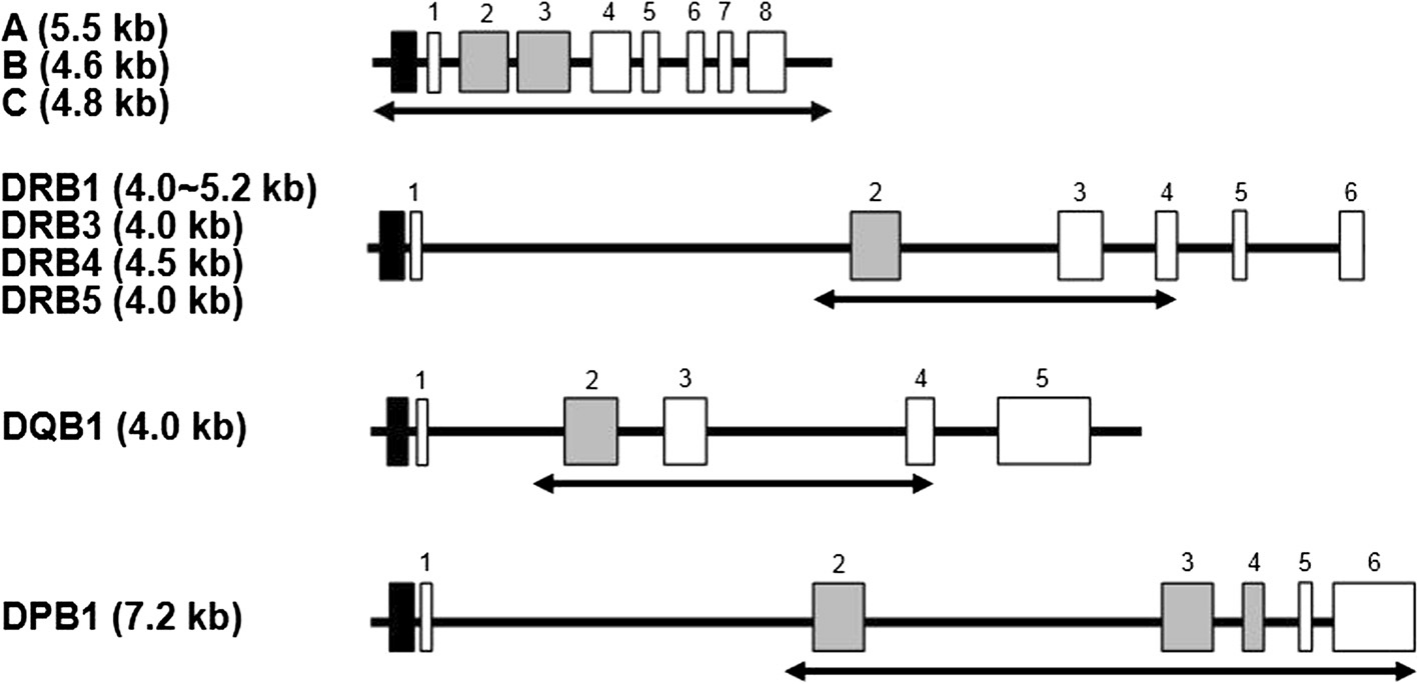
Outline of the targeted PCR regions in nine HLA loci. Black, gray and white boxes indicate promoter regions, highly polymorphic exons and the other exons, respectively.

Although, a few samples like JPN15 (DPB1*05:01:01/DPB1*135:01 and DPB1*25:01) were not fully resolved by the multiplex PCR-NGS method, this problem could be solved in future by determining the full gene nucleotide sequence for the DPB1 gene with the *25:01 allele. Hence, it is necessary to comprehensively collect the HLA allele sequences for all of the PCR regions of all the HLA genes to avoid misidentifying the true locus because of potential problems of allele sharing between different loci or PCR amplification of sequences in the wrong gene regions. In this study, only five ambiguous HLA alleles, DRB1*04:07:01/*04:92, DRB1*04:10:01/*04:10:03, DRB1*09:01:02/*09:21, DRB1*12:01:01/*12:10, and DPB1*13:01/*107:01, were observed at 17 loci. In these cases, the ambiguities were not solved because the informative SNPs for these genes are outside of the PCR regions such as in some of the introns or the 5′ and 3′ non-coding regions or because the informative SNPs that differentiate between ambiguous alleles are located outside of the PCR regions such as within exon 1 or exon 4 of HLA-DRB1 or HLA-DPB1 (Figure 5). It is noteworthy, however, that there was no problem with phase ambiguity for more than 99% of the HLA alleles detected by the 9LOCI method that included the signature sequences of the highly polymorphic exon 2 that play an important role for antigen presentation. Therefore, the multiplex PCR-NGS HLA genotyping method that we have described here is highly effective, accurate and informative and provides an important alternative to the conventional HLA genotyping methods such as SBT and PCR-SSOP that are currently in use in the clinical laboratory. When we applied our previously published long-range PCR primer sets for the ambiguous loci [31], DPB1*05:01:01 and DPB1*25:01, DRB1*04:07:01, DRB1*04:10:03, DRB1*09:01:02, DRB1*12:01:01, and DPB1*13:01 were assigned without ambiguity. These alleles were consistent with known HLA alleles previously assigned by the conventional methods (Additional file 4: Table S3).

To evaluate the sequencing parameters for the 9LOCI PCR-NGS method, we compared the sequencing depth derived from the sequence read numbers between HLA alleles and among HLA loci. The lowest observed average depth (59.2 ± 34.6) was for HLA-DPB1 among the six loci with more than a read depth of 25 (Table 2), where a depth of at least 30 is necessary to identify genetic variants with the highest sensitivity and resolution [38]. In this respect, it will be necessary to improve the sequence reads of HLA-DPB1 by further optimization of primer composition.

In contrast to the one simple multiplex PCR step that is required for the 9 HLA loci, the singleplex PCR models described in Figure 1B (1) and B (2) require many more repetitive steps to amplify the 9 HLA loci and at least five complicated PCR steps for each locus such as preparation of PCR reagents and DNA templates, long range singleplex PCR, electrophoresis, purification, and quantitative determination of the PCR products before preparing the single locus tagging NGS libraries and pooling of all libraries (singleplex PCR/singleplex NGS library model, Figure 1B (1)), and/or before pooling of all PCR products and preparing the multiple locus tagging NGS libraries (singleplex PCR/multiplex NGS library model, Figure 1 (2)). As a more efficient, economical and rapid alternative to the time-consuming singleplex PCR of HLA loci, we developed four types of multiplex PCR methods (CI, CII, 7LOCI, and 9LOCI) for NGS-based HLA genotyping of polymorphic exons.

All of the four types of multiplex PCR methods are useful for the HLA genotyping (this study and data not shown), but the 9LOCI method is likely to be the more valuable method for future routine genotyping for the following reasons and technical advantages. (1) The 9LOCI genotyping method is capable not only for typing the specific HLA loci such as HLA-A, HLA-B, and HLA-DRB1, but also other classical HLA loci such as HLA-C, HLA-DRB3/4/5, HLA-DQB1, and HLA-DPB1 at the same time (Additional file 3: Table S2). (2) The running cost and operation time for the PCR step in the 9LOCI method were reduced to one sixth of the singleplex PCR models. The use of only one micro-tube per sample for the PCR and NGS steps is time-saving and economically helpful for cost-savings on micro-tubes, DNA polymerase and other reagents, and it also eliminates the pooling operations (Figure 1B (3)). If the quantity of all template DNA samples is adjusted accurately among the samples, then electrophoresis of the samples also could be omitted because the PCR products are quantified by the PicoGreen assay after their purification. Thus, omission of some processes such as the pooling step that can influence the sequence read numbers could help to a further reduce potential human experimental errors. (3) The PCR step of multiplex 9LOCI PCR-NGS method for 46 DNA samples was performed in one day. In comparison, the singleplex methods using 46 DNA samples for each of the nine HLA loci would have taken at least three days. (4) Investigation of the template DNA amount suggested that 1 ng of template DNA is sufficient for genotyping all nine HLA loci by the 9LOCI method (Figure 4). The 1 ng amount for the multiplex method is much less than that required for the singleplex PCR methods (Figure 1A (1) and B (2)), and therefore markedly reduced the progressive loss of valuable DNA samples that are required for genotyping of nine HLA loci. This small DNA amount also could be helpful for DNA typing from swab samples derived from oral mucosa cells, FACS derived lymphoma cells and other valuable clinical samples. (5) Therefore, the multiplex PCR method for the nine HLA loci greatly simplifies the procedures required in preparing the DNA samples for NGS by reducing the time of preparation and the amount and costs of reagents, including the use of much smaller amounts of template DNA samples. In addition, the use of different NGS methods might further improve the simplicity and cost of the multiplex PCR-NGS method in the future. For example, a new protocol using Ion Isothermal Amplification Chemistry that enables sequence reads of up to and beyond 500 bp, and Ion Hi-Q™ Sequencing Chemistry that reduces consensus insertion and deletion (indel) errors including homopolymer errors will be available in the near future (personal communication with Life Technologies), and might lead to further simplification and cost reduction with higher data quality.

## Conclusions

Our aim was to simplify and streamline the NGS-based HLA genotyping method as an alternative to the conventional HLA genotyping methods. Although 46 genomic DNA samples were used in the present study as an example of using multiple samples in a single genotyping run, we have recently applied the same methods for genotyping more than 500 DNA samples from Japanese, Indian and French populations in a number of different genotyping runs to unequivocally define the HLA-A, HLA-B, HLA-C, HLA-DRB1/3/4/5, HLA-DQB1, and HLA-DPB1 loci to single HLA alleles to the field 3 level without ambiguity. Therefore, the multiplex PCR methods have contributed greatly to simplify, accelerate and reduce costs and reagents at the PCR step in the NGS-based HLA genotyping method. The methods also conserve on the amounts of DNA samples needed to genotype a multiple number of HLA loci. Overall, the multiplex PCR methods are a powerful tool that provides precise genotyping data without phase ambiguity and with a potential to replace the current routine genotyping methods to find polymorphisms. These methods may help to further activate many fields of medical research involved in the studies of transplantation, disease association, drug adverse reaction, peptide vaccination treatment for cancer and provide us with a better understanding about the diversity and evolution of the human MHC.

## Methods

### Genomic DNA samples

A total of 3,115 donors for bone marrow transplantation through the Japan Marrow Donor Program (JMDP) between 2006 and 2010 were retrospectively genotyped for HLA-A, HLA-B, HLA-C, HLA-DRB1, HLA-DQB1, and HLA-DPB1 alleles to the field 2 level (4-digit typing) as described elsewhere [39]. Of these genotyped donor samples, 46 genomic DNA samples (JPN01 to JPN46) were selected as a reference set based on the distribution of the HLA allele frequency data in the Japanese population (HLA laboratory: http://www.hla.or.jp/haplo/haplonavi.php?type=aril&lang=en) (Additional file 6: Table S5). The reference samples represented more than 99.5% of HLA alleles at each HLA locus with 99.6% at HLA-A, 99.6% at HLA-B, 99.7% at HLA-C, 99.8% at HLA-DRB1, 100% at HLA-DQB1, and 99.9% at HLA-DPB1. In this regard, the reference set included 18 HLA-A alleles, 37 HLA-B alleles, 18 HLA-C alleles, 31 HLA-DRB1 alleles, 14 HLA-DQB1 alleles, and 18 HLA-DPB1 alleles (Additional file 7: Table S6). In addition, approximately 200 genomic DNA samples collected from populations in Africa and Europe were used for an initial study of the optimization of the multiplex PCR methods demonstrating that the method works for various worldwide populations as well as for Japanese. The Japanese HLA genotyping results using the Luminex method are shown in Additional file 4: Table S3. Informed consents were obtained from donors in accordance with the Declaration of Helsinki, and the study protocol was approved from the institutional review board of JMDP and Tokai University.

### PCR primer designation and multiplex PCR amplification

To develop multiplex PCR systems we used previously designed HLA-A, HLA-B, HLA-C, and HLA-DPB1 locus-specific primer sets that cover the whole gene regions from the promoter-enhancer region to 3′UTR with the product size of 5.5 kb in HLA-A, 4.6 kb in HLA-B, and 4.8 kb in HLA-C, and from intron 1 to 3′UTR with the product size of 7.2 kb in HLA-DPB1 [31]. Also, we newly designed an HLA-DRB1/3/4/5 DRB-specific primer set and an HLA-DQB1 locus-specific primer set (available upon request) that cover polymorphic exons (exons 2 and 3) from intron 1 to exon 4 with the product size of 4.0-5.2 kb in HLA-DRB1, 4.1 kb in HLA-DRB3, 4.5 kb in HLA-DRB4, 4.1 kb in HLA-DRB5, and 3.9-4.3 kb in HLA-DQB1 based on the genomic sequences released from GenBank/EMBL/DDBJ DNA databases (accession numbers NG_002392, NG_002433, and NG_002432) and conserved regions of 1000 genome sequences (http://www.1000genomes.org/) (Figure 5). Multiplex PCR methods were constructed using the primer sets by carefully optimizing primer composition and PCR conditions and by comparing to sequence read data from NGS (data not shown).

For PCR amplification of the multiplex PCR methods, the 20 μL PCR amplification-reaction-volume contained 1–25 ng of genomic DNA, 1 unit of PrimeSTAR GXL DNA polymerase (TaKaRa Bio, Shiga, Japan), 4.0 μL of 5 × PrimeSTAR GXL Buffer (5 mM Mg^2+^), 1.6 μL of 2.5 mM of each dNTP and 3.2-5.1 μL (10 pmol/μL) of each primer mixture. The cycling parameters were as follows: primary denaturation 94°C/2 min., followed by 30 cycles for 98°C/10 sec. and 70°C/4 min. The PCR reactions were performed using the thermal cycler GeneAmp PCR system 9700 (Applied Biosystems/Life Technologies/Thermo Fisher Scientific, Foster City, CA). The DNA size was measured by using a 1 kb DNA ladder marker (New England BioLabs, Ipswich, MA). The PCR products were purified by the Agencourt AMPure XP (Beckman Coulter, Fullerton, CA) and quantified by the PicoGreen assay (Invitrogen/Life Technologies/Thermo Fisher Scientific, Carlsbad, CA) with a Fluoroskan Ascent micro-plate fluorometer (Thermo Fisher Scientific, Waltham, MA).

### NGS using Ion Torrent PGM system

Barcoded-library DNA samples were prepared with an Ion Xpress Plus Fragment Library Kit and Ion Xpress barcode Adaptors 1–96 Kit according to the manufacturer’s protocol for 400 base-read sequencing (Life Technologies/Thermo Fisher Scientific, Palo Alto, CA). One hundred nanograms of the multiplex PCR products were used for the preparation of each DNA library. DNA samples were fragmented with a M220 Focused-ultrasonicator (Covaris, Woburn, MA). Each DNA library was amplified by eight cycles of PCR. The DNA size and quantitation for each library was measured by an Agilent 2100 Expert Bioanalyzer using the Agilent High Sensitivity DNA Kit (Agilent Technologies, Santa Clara, CA). Each barcoded-library was mixed at equimolar concentrations then diluted according to the manufacturer’s recommendation. Emulsion PCR (emPCR) was performed using the resulting pooled library with the Ion PGM Template OT2 400 Kit on an Ion OneTouch 2 automated system (Life Technologies) with the following cycling parameters: primary denaturation 95°C/10 min., followed by 20 cycles for 95°C/30 sec., 66°C/4 min., 20 cycles for 95°C/30 sec., 66°C/6 min. and 10 cycles for 95°C/30 sec., and 66°C/20 min. After the emulsion was automatically broken with the OneTouch 2 instrument, the beads carrying the single-stranded DNA templates were enriched according to the manufacturer’s recommendation. Sequencing was performed using the Ion PGM Sequencing 400 Kit and Ion 316 and 318 Chip Kit v2 with a flow number of 850 for 400 base-read [40].

### Data processing and allele assignment

The raw data processing and base-calling, trimming and output of quality-filter sequence reads that were binned on the basis of the Ion Xpress Barcodes into 46 separate sequence fastq files, were all performed by the Torrent Suite 4.2.1 software (Life Technologies) with full processing for shotgun analysis. These files were further quality trimmed to remove poor sequence at the end of the reads with QVs of less than 10. The trimmed and barcode-binned sequence reads were used for HLA allele assignment to the field 3 and 4 levels by Sequence Alignment Based Assigning Software (SeaBass) (an in-house development of Tokai University, in preparation). HLA allele candidates and/or reference sequences used for mapping of the sequence reads were selected by nucleotide similarity searches with HLA allele sequences in the IMGT-HLA database using the BLAT program (http://genome.ucsc.edu/), and thereafter, mapping of the sequence reads and the selected reference sequences were performed automatically with the GS Reference Mapper Ver. 3.0 software (Life Technologies). The mapping parameter was set to a perfectly matched condition between the read sequences and the reference sequences to avoid mismapping among the HLA loci and contamination of *in vitro* generated PCR crossover products [41]. If a reference sequence covering the PCR region was not available, we constructed a new virtual sequence by *de novo* assembly using the Sequencher Ver. 5.0.1 DNA sequence assembly software (Gene Code, Ann Arbor, MI), and used it as a reference sequence.

### Calculation of uniformity among HLA loci and between HLA alleles

After assignment of the HLA alleles we calculated uniformity among the HLA loci and between alleles using the sequence reads that separated to each allele. The read depth is the number of individual sequence reads that align to a particular nucleotide position [25]. An average depth of exons 2 and 3 in class I loci, HLA-A, HLA-B, and HLA-C, and exon 2 in class II loci, HLA-DRB1, HLA-DQB1, and HLA-DPB1, was calculated as an average redundancy per nucleotide site (the sum of depth on all nucleotide site numbers/nucleotide site numbers). Average depth ratio was calculated as an average depth of one allele/large average depth of the other allele. Depth per locus was calculated by the sum of average depth of both alleles.

## Additional files

Additional file 1: Figure S1. Electrophoresis images of PCR products from 46 genomic DNA samples JPN01 to JPN46 using the 9LOCI method. The short description of the data: Electrophoresis images of PCR products from 46 genomic DNA samples (JPN01 to JPN46) were shown in the figure. The HLA loci in the bands amplified by PCR are indicated on the right side of the figure. Additional file 2: Table S1. Sequence read information obtained by the Ion PGM system. The short description of the data: Draft read numbers, draft read bases, average read length, mode read length, and average quality value of sequence reads derived from 46 DNA samples. Additional file 3: Table S2. Alleles for nine HLA loci obtained by the 9LOCI genotyping method. The short description of the data: Genotypes of nine HLA loci obtained by the 9LOCI method. Additional file 4: Table S3. HLA alleles for 46 Japanese DNA samples using the Luminex genotyping method. The short description of the data: A list of HLA genotypes of 46 DNA samples used in this study. Additional file 5: Table S4. Depth information of each allele and each locus. The short description of the data: Genotypes and average depth, average depth ratio, and depth per loci of 276 loci obtained by the 9LOCI method. Additional file 6: Table S5. HLA allele frequency data in Japanese population. The short description of the data: A list of the HLA allele frequencies and ranking in the Japanese population. Additional file 7: Table S6. The number and frequency of HLA alleles in the Japanese population that were genotyped for the 46 Japanese reference DNA samples used in this study. The short description of the data: A list of HLA alleles frequencies in the Japanese population including the 46 DNA samples used in this study.

## Abbreviations

BLAT: BLAST-like alignment tool
FACS: Fluorescence activated cell sorting
GVHD: Graft-versus-host disease
HLA: Human leukocyte antigen
Indel: Insertion and deletion
MHC: Major histocompatibility complex
NGS: Next generation sequencing
PCR: Polymerase chain reaction
QV: Quality value
RFLP: Restriction fragment polymorphisms
SBT: Sequence based typing
SD: Standard deviation
SeaBass: Sequence alignment based assigning software
SMRT: Single molecule real-time
SSCP: Single strand conformation polymorphism
SSOP: Sequence specific oligonucleotide primers
SSOs: Sequence specific oligonucleotides
SSPs: Sequence specific primers
SS-SBT: Super high resolution-single molecule-sequence-based typing
