# Supplementary figures and images for: Cost-efficient multiplex PCR for routine genotyping of up to nine classical HLA loci in a single analytical run of multiple samples by next generation sequencing
Source: BMC Genomics. 2015 Apr 18;16(1):318. doi: 10.1186/s12864-015-1514-4 (PMC4404632; doi:10.1186/s12864-015-1514-4)

Figure S1

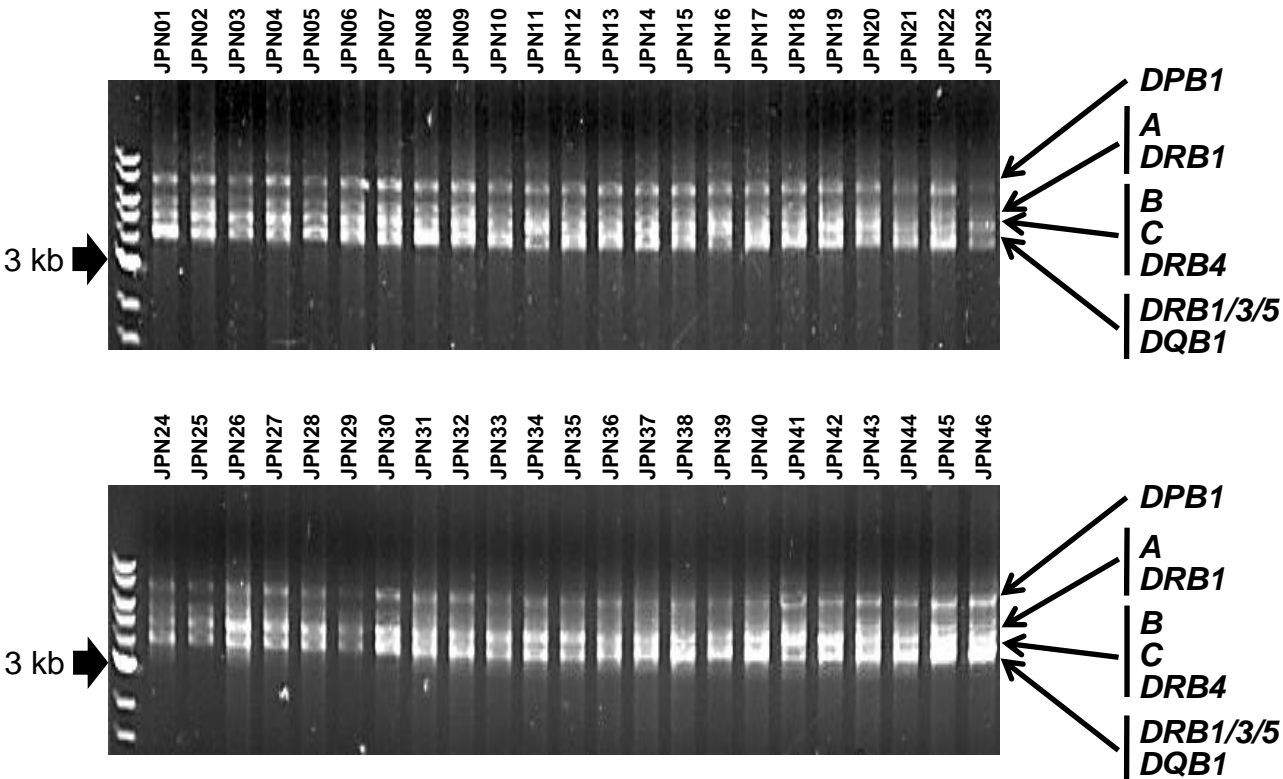

Supplement: Additional file 1: Figure S1. — Electrophoresis images of PCR products from 46 genomic DNA samples JPN01 to JPN46 using the 9LOCI method. The short description of the data: Electrophoresis images of PCR products from 46 genomic DNA samples (JPN01 to JPN46) were shown in the figure. The HLA loci in the bands amplified by PCR are indicated on the right side of the figure. [file 12864_2015_1514_MOESM1_ESM.pdf]
