# Supplementary material for: Cost-efficient multiplex PCR for routine genotyping of up to nine classical HLA loci in a single analytical run of multiple samples by next generation sequencing
Source: BMC Genomics. 2015 Apr 18;16(1):318. doi: 10.1186/s12864-015-1514-4 (PMC4404632; doi:10.1186/s12864-015-1514-4)
Supplement: Additional file 2: Table S1. — Sequence read information obtained by the Ion PGM system. The short description of the data: Draft read numbers, draft read bases, average read length, mode read length, and average quality value of sequence reads derived from 46 DNA samples. [file 12864_2015_1514_MOESM2_ESM.pdf]

Table S1. Sequence read information obtained by the Ion PGM system

DNA sample ID	Draft read numbers	Draft read bases (b)	Average read length (b)	Mode read length (b)	Average quality value
JPN01	102,924	26,777,089	260.2	333.0	28.3
JPN02	115,395	31,396,613	272.1	348.0	28.2
JPN03	88,274	24,146,819	273.5	349.0	28.2
JPN04	111,802	31,726,851	283.8	362.0	28.0
JPN05	91,753	24,857,331	270.9	352.0	28.2
JPN06	99,354	26,724,631	269.0	352.0	28.0
JPN07	99,795	26,645,323	267.0	340.0	28.2
JPN08	95,078	22,595,570	237.7	333.0	28.2
JPN09	101,360	27,492,820	271.2	351.0	28.2
JPN10	84,387	22,928,751	271.7	362.0	27.9
JPN11	93,856	25,559,951	272.3	366.0	27.9
JPN12	95,108	27,386,052	287.9	373.0	27.9
JPN13	127,234	33,359,933	262.2	334.0	28.2
JPN14	134,709	36,083,290	267.9	340.0	28.2
JPN15	119,701	33,046,257	276.1	356.0	28.0
JPN16	139,593	39,330,548	281.8	361.0	27.9
JPN17	99,654	27,837,179	279.3	363.0	27.9
JPN18	114,545	32,549,231	284.2	361.0	27.7
JPN19	114,463	31,581,043	275.9	357.0	28.1
JPN20	118,674	32,435,151	273.3	361.0	28.0
JPN21	83,680	21,787,420	260.4	372.0	27.8
JPN22	125,040	34,100,076	272.7	372.0	27.9
JPN23	124,995	32,515,813	260.1	361.0	28.2
JPN24	115,888	32,682,571	282.0	361.0	28.0
JPN25	91,573	23,481,465	256.4	329.0	28.2
JPN26	96,482	26,008,022	269.6	340.0	28.2
JPN27	112,634	31,147,131	276.5	349.0	28.2
JPN28	103,440	29,427,161	284.5	361.0	28.0
JPN29	94,919	26,825,990	282.6	361.0	28.0
JPN30	96,811	26,916,678	278.0	350.0	28.2
JPN31	102,403	27,382,583	267.4	376.0	27.9
JPN32	115,711	31,364,124	271.1	380.0	27.8
JPN33	85,879	22,436,090	261.3	373.0	28.0
JPN34	153,990	42,009,365	272.8	384.0	27.7
JPN35	125,981	36,530,500	290.0	395.0	27.7
JPN36	136,448	38,294,945	280.7	385.0	27.9
JPN37	136,123	36,493,268	268.1	323.0	28.1
JPN38	153,560	42,040,422	273.8	348.0	28.2
JPN39	108,283	29,385,880	271.4	348.0	28.1
JPN40	142,540	39,813,312	279.3	370.0	27.9
JPN41	152,925	43,422,500	283.9	381.0	27.7
JPN42	119,540	35,023,478	293.0	397.0	27.6
JPN43	135,485	36,115,139	266.6	348.0	28.0
JPN44	123,562	33,752,065	273.2	348.0	28.2
JPN45	156,157	42,788,174	274.0	363.0	28.0
JPN46	142,862	40,388,264	282.7	368.0	27.9
Total	5,284,570	1,446,592,869			
Average	114,882	31,447,671	273.3	358.6	28.0
±SD	20,482	5,990,141	9.9	16.7	0.2
Maximum value	156,157	43,422,500	293.0	397.0	28.3
Minimum value	83,680	21,787,420	237.7	323.0	27.6
