# Supplementary material for: Cost-efficient multiplex PCR for routine genotyping of up to nine classical HLA loci in a single analytical run of multiple samples by next generation sequencing
Source: BMC Genomics. 2015 Apr 18;16(1):318. doi: 10.1186/s12864-015-1514-4 (PMC4404632; doi:10.1186/s12864-015-1514-4)
Supplement: Additional file 3: Table S2. — Alleles for nine HLA loci obtained by the 9LOCI genotyping method. The short description of the data: Genotypes of nine HLA loci obtained by the 9LOCI method. [file 12864_2015_1514_MOESM3_ESM.pdf]

Table S2. Alleles for nine HLA loci obtained by the 9LOCI genotyping method

sample ID	HLA-A		HLA-B		HLA-C		HLA-DRB1		HLA-DRB3/4/5		HLA-DQB1		HLA-DPB1	
	Allele 1	Allele 2	Allele 1	Allele 2	Allele 1	Allele 2	Allele 1	Allele 2	Allele 1	Allele 2	Allele 1	Allele 2	Allele 1	Allele 2
JPN01	A*02:06:01	A*31:01:02	B*39:01:01	B*40:02:01	C*03:04:01	C*07:02:01	DRB1*08:03:02	DRB1*14:54:01	DRB3*02:02:01	-	DQB1*05:02:01	DQB1*06:01:01	DPB1*04:02:01	DPB1*05:01:01
JPN02	A*24:02:01	-	B*07:02:01	B*55:02:01	C*01:02:01	C*07:02:01	DRB1*01:01:01	DRB1*04:06:01	DRB4*01:03:02	-	DQB1*03:02:01	DQB1*05:01:01	DPB1*02:01:02	DPB1*13:01/*107:01
JPN03	A*02:01:01	A*31:01:02	B*40:06:01	B*48:01:01	C*08:01:01	C*08:03:01	DRB1*09:01:02/*09:21	-	DRB4*01:03:02	-	DQB1*03:03:02	-	DPB1*05:01:01	-
JPN04	A*11:01:01	A*24:02:01	B*15:01:01	B*55:02:01	C*08:01:01	C*12:03:01	DRB1*04:05:01	DRB1*09:01:02/*09:21	DRB4*01:03:01	-	DQB1*03:03:02	DQB1*04:01:01	DPB1*02:02	-
JPN05	A*02:01:01	A*02:06:01	B*35:01:01	B*51:02:01	C*03:03:01	C*15:02:01	DRB1*08:02:01	DRB1*11:01:01	DRB3*02:02:01	-	DQB1*03:01:01	DQB1*04:02:01	DPB1*04:02:01	DPB1*05:01:01
JPN06	A*26:01:01	A*33:03:01	B*40:02:01	B*44:03:01	C*03:04:01	C*14:03	DRB1*04:05:01	DRB1*13:02:01	DRB3*03:01:01	DRB4*01:03:01	DQB1*04:01:01	DQB1*06:04:01	DPB1*02:01:02	DPB1*04:01:01
JPN07	A*24:02:01	-	B*07:02:01	B*15:07:01	C*03:03:01	C*07:02:01	DRB1*01:01:01	DRB1*04:06:01	DRB4*01:03:01	-	DQB1*03:02:01	DQB1*05:01:01	DPB1*02:01:02	DPB1*04:02:01
JPN08	A*24:02:01	-	B*13:01:01	B*40:03	C*03:04:01	-	DRB1*12:01:01/*12:10	DRB1*12:02:01	DRB3*01:01:02	DRB3*03:01:03	DQB1*03:01:01	-	DPB1*02:01:02	DPB1*05:01:01
JPN09	A*11:01:01	A*24:02:01	B*35:01:01	B*56:01:01	C*01:02:01	C*01:02:01	DRB1*04:05:01	DRB1*11:01:01	DRB3*02:02:01	DRB4*01:03:01	DQB1*03:01:01	DQB1*04:01:01	DPB1*04:02:01	DPB1*05:01:01
JPN10	A*24:02:01	A*33:03:01	B*07:02:01	B*58:01:01	C*03:02:02	C*07:02:01	DRB1*01:01:01	DRB1*13:02:01	DRB3*03:01:01	-	DQB1*05:01:01	DQB1*06:09:01	DPB1*02:01:02	DPB1*05:01:01
JPN11	A*24:02:01	A*26:01:01	B*48:01:01	B*54:01:01	C*01:02:01	C*08:01:01	DRB1*04:05:01	DRB1*04:07:01/*04:92	DRB4*01:03:01	-	DQB1*03:02:01	DQB1*04:01:01	DPB1*02:01:02	DPB1*19:01
JPN12	A*03:01:01	A*24:02:01	B*07:02:01	B*44:02:01	C*05:01:01	C*07:02:01	DRB1*01:01:01	DRB1*13:01:01	DRB3*01:01:02	-	DQB1*05:01:01	DQB1*06:03:01	DPB1*04:02:01	-
JPN13	A*02:06:01	A*02:10:01	B*15:01:01	B*40:06:01	C*03:04:01	C*08:01:01	DRB1*12:02:01	DRB1*15:01:01	DRB3*03:01:03	DRB5*01:01:01	DQB1*03:01:01	DQB1*06:02:01	DPB1*05:01:01	-
JPN14	A*01:01:01	A*24:02:01	B*37:01:01	B*51:01:01	C*06:02:01	C*14:02:01	DRB1*10:01:01	DRB1*14:03:01	DRB3*01:01:02	-	DQB1*03:01:01	DQB1*05:01:01	DPB1*02:01:02	-
JPN15	A*02:06:01	A*26:01:01	B*35:01:01	B*40:02:01	C*03:03:01	C*03:04:01	DRB1*04:05:01	DRB1*11:01:01	DRB3*02:02:01	DRB4*01:03:02	DQB1*03:01:01	DQB1*04:01:01	DPB1*05:01/*135:01	DPB1*25:01
JPN16	A*02:01:01	A*24:02:01	B*15:18:01	B*35:01:01	C*03:03:01	C*07:04-new	DRB1*04:01:01	DRB1*04:10:01/*04:10:03	DRB4*01:02	DRB4*01:03:01	DQB1*03:01:01	DQB1*04:02:01	DPB1*02:01:02	DPB1*14:01
JPN17	A*24:02:01	A*31:01:02	B*51:01:01	B*52:01:01	C*12:02:02	C*14:02:01	DRB1*14:05:01	DRB1*15:02:01	DRB3*02:02:01	DRB5*01:02	DQB1*05:03:01	DQB1*06:01:01	DPB1*09:01:01	DPB1*14:01
JPN18	A*02:06:01	A*31:01:02	B*39:01:03	B*40:02:01	C*03:04:01	C*07:02:01	DRB1*08:02:01	DRB1*12:02:01	DRB3*03:01:03	-	DQB1*03:01:01	DQB1*04:02:01	DPB1*02:01:02	DPB1*06:01
JPN19	A*24:02:01	-	B*07:02:01	B*39:04	C*07:02:01	-	DRB1*01:01:01	DRB1*09:01:02/*09:21	DRB4*01:03:02	-	DQB1*03:03:02	DQB1*05:01:01	DPB1*04:02:01	DPB1*05:01:01
JPN20	A*02:06:01	A*33:03:01	B*58:01:01	B*59:01:01	C*01:02:01	C*03:02:02	DRB1*03:01:01	DRB1*04:05:01	DRB3*02:02:01	DRB4*01:03:01	DQB1*02:01:01	DQB1*04:01:01	DPB1*04:02:01	DPB1*05:01:01
JPN21	A*02:01:01	A*11:01:01	B*35:01:01	B*56:03	C*01:02:01	C*03:03:01	DRB1*12:01:01/*12:10	DRB1*15:01:01	DRB3*01:01:02	DRB5*01:01:01	DQB1*03:01:01	DQB1*06:02:01	DPB1*02:01:02	DPB1*14:01
JPN22	A*02:07:01	A*11:02:01	B*27:04:01	B*46:01:01	C*01:02:01	C*12:02:02	DRB1*08:03:02	DRB1*12:01:01/*12:10	DRB3*01:01:02	-	DQB1*03:01:01	DQB1*06:01:01	DPB1*05:01:01	-
JPN23	A*02:07:01	A*11:01:01	B*46:01:01	-	C*01:02:01	C*01:03	DRB1*08:03:02	-	-	-	DQB1*06:01:01	-	DPB1*02:01:02	DPB1*05:01:01
JPN24	A*11:01:01	A*24:02:01	B*35:01:01	B*39:01:01	C*03:03:01	C*07:02:01	DRB1*08:03:02	DRB1*15:01:01	DRB5*01:01:01	-	DQB1*06:01:01	DQB1*06:02:01	DPB1*05:01:01	DPB1*38:01
JPN25	A*11:01:01	A*24:02:01	B*15:01:01	B*38:02:01	C*04:01:01	C*07:02:01	DRB1*04:05:01	DRB1*08:03:02	DRB4*01:03:01	-	DQB1*03:01:01	DQB1*04:01:01	DPB1*05:01:01	DPB1*14:01
JPN26	A*24:02:01	-	B*15:01:01	B*15:27:01	C*04:01:01	-	DRB1*04:06:01	DRB1*09:01:02/*09:21	DRB4*01:03:01	-	DQB1*03:02:01	DQB1*03:03:02	DPB1*02:01:02	DPB1*14:01
JPN27	A*02:01:01	A*26:01:01	B*40:01:02	B*54:01:01	C*01:02:01	C*07:02:01	DRB1*04:05:01	DRB1*08:09	DRB4*01:03:01	-	DQB1*04:01:01	DQB1*04:02:01	DPB1*05:01:01	-
JPN28	A*02:01:01	A*26:03:01	B*15:01:01	B*46:01:01	C*01:03	C*03:03:01	DRB1*09:01:02/*09:21	-	DRB4*01:03:02	-	DQB1*03:03:02	-	DPB1*05:01:01	-
JPN29	A*02:01:01	A*11:01:01	B*35:01:01	B*40:02:01	C*03:03:01	-	DRB1*04:05:01	DRB1*15:01:01	DRB4*01:03:02	DRB5*01:01:01	DQB1*04:01:01	DQB1*06:02:01	DPB1*02:01:02	DPB1*47:01
JPN30	A*02:01:01	A*24:02:01	B*52:01:01	B*55:04	C*03:03:01	C*12:02:02	DRB1*09:01/21	DRB1*15:02:01	DRB4*01:03:02	DRB5*01:02	DQB1*03:03:02	DQB1*06:01:01	DPB1*02:01:02	DPB1*05:01:01
JPN31	A*02:01:01	A*26:02:01	B*35:01:01	B*40:01:02	C*03:03:01	C*07:02:01	DRB1*04:05:01	DRB1*12:01:01/*12:10	DRB3*01:12	DRB4*01:03:01	DQB1*03:01:01	DQB1*04:01:01	DPB1*02:01:02	DPB1*36:01
JPN32	A*02:01:01	A*24:02:01	B*35:01:01	B*39:02:01	C*03:03:01	C*07:02:01	DRB1*08:03:02	DRB1*09:01:02/*09:21	DRB4*01:03:02	-	DQB1*03:03:02	DQB1*06:01:01	DPB1*02:01:02	-
JPN33	A*02:01:01	A*11:01:01	B*15:01:01	B*15:11:01	C*03:03:01	C*04:01:01	DRB1*04:06:01	DRB1*09:01:02/*09:21	DRB4*01:03:01	DRB4*01:03:02	DQB1*03:02:01	DQB1*03:03:02	DPB1*02:01:02	DPB1*05:01:01
JPN34	A*02:01:01	A*24:02:01	B*15:01:01	B*40:50	C*03:04:01	C*04:01:01	DRB1*04:06:01	DRB1*08:02:01	DRB4*01:03:01	-	DQB1*03:02:01	-	DPB1*02:01:02	DPB1*05:01:01
JPN35	A*02:18	A*11:01:01	B*15:01:01	B*46:01:01	C*01:02:01	C*04:01:01	DRB1*04:06:01	DRB1*08:03:02	DRB4*01:03:01	-	DQB1*03:02:01	DQB1*06:01:01	DPB1*02:02	DPB1*05:01:01
JPN36	A*24:02:01	A*30:01:01	B*13:02:01	B*51:01:01	C*06:02:01	C*14:02:01	DRB1*07:01:01	DRB1*14:03:01	DRB3*01:01:02	DRB4*01:03:01	DQB1*02:02:01	DQB1*03:01:01	DPB1*17:01	DPB1*41:01:01
JPN37	A*03:02:01	A*24:02:01	B*13:02:01	B*40:06:01	C*03:03:01	C*06:02:01	DRB1*07:01:01	DRB1*12:01:01/*12:10	DRB3*01:01:02	DRB4*01:03:01	DQB1*02:02:01	DQB1*03:01:01	DPB1*05:01:01	DPB1*17:01
JPN38	A*02:01:01	A*31:01:02	B*40:02:01	B*51:01:01	C*03:04:01	C*14:02:01	DRB1*08:02:01	DRB1*14:02:01	DRB3*02:02:01	-	DQB1*03:01:01	DQB1*04:02:01	DPB1*02:02	DPB1*05:01:01
JPN39	A*24:02:01	A*31:01:02	B*39:23	B*52:01:01	C*07:02:01	C*12:02:02	DRB1*14:06:01	DRB1*15:02:01	DRB3*02:02:01	DRB5*01:02	DQB1*03:01:01	DQB1*06:01:01	DPB1*05:01:01	DPB1*09:01:01
JPN40	A*24:02:01	A*24:20:01	B*07:02:01	B*13:01:01	C*03:04:01	C*07:02:01	DRB1*01:01:01	DRB1*14:07:01	DRB3*02:02:01	-	DQB1*05:01:01	DQB1*05:03:01	DPB1*04:02:01	DPB1*05:01:01
JPN41	A*02:01:01	A*31:01:02	B*39:01:03	B*40:01:02	C*03:04:01	C*07:02:01	DRB1*04:03:01	DRB1*04:04:01	DRB4*01:03:01	-	DQB1*03:02:01	-	DPB1*02:01:02	-
JPN42	A*24:02:01	A*26:01:01	B*40:01:02	B*40:02:01	C*03:04:01	C*03:23:01	DRB1*09:01:02/*09:21	-	DRB4*01:03:02	-	DQB1*03:03:02	-	DPB1*02:01:02	DPB1*05:01:01
JPN43	A*11:01:01	-	B*15:01:01	B*67:01:01	C*04:01:01	C*07:02:01	DRB1*04:06:01	DRB1*16:02:01	DRB4*01:03:01	DRB5*02:02	DQB1*03:02:01	DQB1*05:02:01	DPB1*02:02	DPB1*48:01
JPN44	A*02:06:01	A*24:02:01	B*27:05:02	B*52:01:01	C*01:02:01	C*12:02:02	DRB1*01:01:01	DRB1*15:02:01	DRB5*01:02	-	DQB1*05:01:01	DQB1*06:01:01	DPB1*04:02:01	DPB1*09:01:01
JPN45	A*24:02:01	-	B*15:18:01	B*52:01:01	C*08:01:01	C*12:02:02	DRB1*13:07:01	DRB1*15:02:01	DRB3*02:02:01	DRB5*01:02	DQB1*03:01:01	DQB1*06:01:01	DPB1*03:01:01	DPB1*09:01:01
JPN46	A*02:01:01	A*24:02:01	B*07:02:01	B*35:01:01	C*07:02:01	C*08:01:01	DRB1*01:01:01	DRB1*11:19:01	DRB3*02:02:01	-	DQB1*03:01:01	DQB1*05:01:01	DPB1*04:02:01	-
