# Supplementary material for: Cost-efficient multiplex PCR for routine genotyping of up to nine classical HLA loci in a single analytical run of multiple samples by next generation sequencing
Source: BMC Genomics. 2015 Apr 18;16(1):318. doi: 10.1186/s12864-015-1514-4 (PMC4404632; doi:10.1186/s12864-015-1514-4)
Supplement: Additional file 4: Table S3. — HLA alleles for 46 Japanese DNA samples using the Luminex genotyping method. The short description of the data: A list of HLA genotypes of 46 DNA samples used in this study. [file 12864_2015_1514_MOESM4_ESM.pdf]

Table S3. HLA alleles for 46 Japanese DNA samples using the Luminex genotyping method

Simple Num.	HLA-A		HLA-B		HLA-C		HLA-DRB1		HLA-DQB1		HLA-DPB1	
	allele 1	allele 2	allele 1	allele 2	allele 1	allele 2	allele 1	allele 2	allele 1	allele 2	allele 1	allele 2
JPN01	A*02:06	A*31:01	B*39:01	B*40:02	C*03:04	C*07:02	DRB1*08:03	DRB1*14:54	DQB1*05:02	DQB1*06:01	DPB1*04:02	DPB1*05:01
JPN02	A*24:02	A*24:02	B*07:02	B*55:02	C*01:02	C*07:02	DRB1*01:01	DRB1*04:06	DQB1*03:02	DQB1*05:01	DPB1*02:01	DPB1*13:01
JPN03	A*02:01	A*31:01	B*40:06	B*48:01	C*08:01	C*08:03	DRB1*09:01	DRB1*09:01	DQB1*03:03	DQB1*03:03	DPB1*05:01	DPB1*05:01
JPN04	A*11:01	A*24:02	B*15:01	B*55:02	C*08:01	C*12:03	DRB1*04:05	DRB1*09:01	DQB1*03:03	DQB1*04:01	DPB1*02:02	DPB1*02:02
JPN05	A*02:01	A*02:06	B*35:01	B*51:02	C*03:03	C*15:02	DRB1*08:02	DRB1*11:01	DQB1*03:01	DQB1*04:02	DPB1*04:02	DPB1*05:01
JPN06	A*26:01	A*33:03	B*40:02	B*44:03	C*03:04	C*14:03	DRB1*04:05	DRB1*13:02	DQB1*04:01	DQB1*06:04	DPB1*02:01	DPB1*04:01
JPN07	A*24:02	A*24:02	B*07:02	B*15:07	C*03:03	C*07:02	DRB1*01:01	DRB1*04:06	DQB1*03:02	DQB1*05:01	DPB1*02:01	DPB1*04:02
JPN08	A*24:02	A*24:02	B*13:01	B*40:03	C*03:04	C*03:04	DRB1*12:01	DRB1*12:02	DQB1*03:01	DQB1*03:01	DPB1*02:01	DPB1*05:01
JPN09	A*11:01	A*24:02	B*35:01	B*56:01	C*01:02	C*01:02	DRB1*04:05	DRB1*11:01	DQB1*03:01	DQB1*04:01	DPB1*04:02	DPB1*05:01
JPN10	A*24:02	A*33:03	B*07:02	B*58:01	C*03:02	C*07:02	DRB1*01:01	DRB1*13:02	DQB1*05:01	DQB1*06:09	DPB1*02:01	DPB1*05:01
JPN11	A*24:02	A*26:01	B*48:01	B*54:01	C*01:02	C*08:01	DRB1*04:05	DRB1*04:07	DQB1*03:02	DQB1*04:01	DPB1*02:01	DPB1*19:01
JPN12	A*03:01	A*24:02	B*07:02	B*44:02	C*05:01	C*07:02	DRB1*01:01	DRB1*13:01	DQB1*05:01	DQB1*06:03	DPB1*04:02	DPB1*04:02
JPN13	A*02:06	A*02:10	B*15:01	B*40:06	C*03:04	C*08:01	DRB1*12:02	DRB1*15:01	DQB1*03:01	DQB1*06:02	DPB1*05:01	DPB1*05:01
JPN14	A*01:01	A*24:02	B*37:01	B*51:01	C*06:02	C*14:02	DRB1*10:01	DRB1*14:03	DQB1*03:01	DQB1*05:01	DPB1*02:01	DPB1*02:01
JPN15	A*02:06	A*26:01	B*35:01	B*40:02	C*03:03	C*03:04	DRB1*04:05	DRB1*11:01	DQB1*03:01	DQB1*04:01	DPB1*05:01	DPB1*25:01
JPN16	A*02:01	A*24:02	B*15:18	B*35:01	C*03:03	C*07:04	DRB1*04:01	DRB1*04:10	DQB1*03:01	DQB1*04:02	DPB1*02:01	DPB1*14:01
JPN17	A*24:02	A*31:01	B*51:01	B*52:01	C*12:02	C*14:02	DRB1*14:05	DRB1*15:02	DQB1*05:03	DQB1*06:01	DPB1*09:01	DPB1*14:01
JPN18	A*02:06	A*31:01	B*39:01	B*40:02	C*03:04	C*07:02	DRB1*08:02	DRB1*12:02	DQB1*03:01	DQB1*04:02	DPB1*02:01	DPB1*06:01
JPN19	A*24:02	A*24:02	B*07:02	B*39:04	C*07:02	C*07:02	DRB1*01:01	DRB1*09:01	DQB1*03:03	DQB1*05:01	DPB1*04:02	DPB1*05:01
JPN20	A*02:06	A*33:03	B*58:01	B*59:01	C*01:02	C*03:02	DRB1*03:01	DRB1*04:05	DQB1*02	DQB1*04:01	DPB1*04:02	DPB1*05:01
JPN21	A*02:01	A*11:01	B*35:01	B*56:03	C*01:02	C*03:03	DRB1*12:01	DRB1*15:01	DQB1*03:01	DQB1*06:02	DPB1*02:01	DPB1*14:01
JPN22	A*02:07	A*11:02	B*27:04	B*46:01	C*01:02	C*12:02	DRB1*08:03	DRB1*12:01	DQB1*03:01	DQB1*06:01	DPB1*05:01	DPB1*05:01
JPN23	A*02:07	A*11:01	B*46:01	B*46:01	C*01:02	C*01:03	DRB1*08:03	DRB1*08:03	DQB1*06:01	DQB1*06:01	DPB1*02:01	DPB1*05:01
JPN24	A*11:01	A*24:02	B*35:01	B*39:01	C*03:03	C*07:02	DRB1*08:03	DRB1*15:01	DQB1*06:01	DQB1*06:02	DPB1*05:01	DPB1*38:01
JPN25	A*11:01	A*24:02	B*15:01	B*38:02	C*04:01	C*07:02	DRB1*04:05	DRB1*08:03	DQB1*03:01	DQB1*04:01	DPB1*05:01	DPB1*14:01
JPN26	A*24:02	A*24:02	B*15:01	B*15:27	C*04:01	C*04:01	DRB1*04:06	DRB1*09:01	DQB1*03:02	DQB1*03:03	DPB1*02:01	DPB1*14:01
JPN27	A*02:01	A*26:01	B*40:01	B*54:01	C*01:02	C*07:02	DRB1*04:05	DRB1*08:09	DQB1*04:01	DQB1*04:02	DPB1*05:01	DPB1*05:01
JPN28	A*02:01	A*26:03	B*15:01	B*46:01	C*01:03	C*03:03	DRB1*09:01	DRB1*09:01	DQB1*03:03	DQB1*03:03	DPB1*05:01	DPB1*05:01
JPN29	A*02:01	A*11:01	B*35:01	B*40:02	C*03:03	C*03:03	DRB1*04:05	DRB1*15:01	DQB1*04:01	DQB1*06:02	DPB1*02:01	DPB1*47:01
JPN30	A*02:01	A*24:02	B*52:01	B*55:04	C*03:03	C*12:02	DRB1*09:01	DRB1*15:02	DQB1*03:03	DQB1*06:01	DPB1*02:01	DPB1*05:01
JPN31	A*02:01	A*26:02	B*35:01	B*40:01	C*03:03	C*07:02	DRB1*04:05	DRB1*12:01	DQB1*03:01	DQB1*04:01	DPB1*02:01	DPB1*36:01
JPN32	A*02:01	A*24:02	B*35:01	B*39:02	C*03:03	C*07:02	DRB1*08:03	DRB1*09:01	DQB1*03:03	DQB1*06:01	DPB1*02:01	DPB1*02:01
JPN33	A*02:01	A*11:01	B*15:01	B*15:11	C*03:03	C*04:01	DRB1*04:06	DRB1*09:01	DQB1*03:02	DQB1*03:03	DPB1*02:01	DPB1*05:01
JPN34	A*02:01	A*24:02	B*15:01	B*40:50	C*03:04	C*04:01	DRB1*04:06	DRB1*08:02	DQB1*03:02	DQB1*03:02	DPB1*02:01	DPB1*05:01
JPN35	A*02:18	A*11:01	B*15:01	B*46:01	C*01:02	C*04:01	DRB1*04:06	DRB1*08:03	DQB1*03:02	DQB1*06:01	DPB1*02:02	DPB1*05:01
JPN36	A*24:02	A*30:01	B*13:02	B*51:01	C*06:02	C*14:02	DRB1*07:01	DRB1*14:03	DQB1*02	DQB1*03:01	DPB1*17:01	DPB1*41:01
JPN37	A*03:02	A*24:02	B*13:02	B*40:06	C*03:03	C*06:02	DRB1*07:01	DRB1*12:01	DQB1*02	DQB1*03:01	DPB1*05:01	DPB1*17:01
JPN38	A*02:01	A*31:01	B*40:02	B*51:01	C*03:04	C*14:02	DRB1*08:02	DRB1*14:02	DQB1*03:01	DQB1*04:02	DPB1*02:02	DPB1*05:01
JPN39	A*24:02	A*31:01	B*39:23	B*52:01	C*07:02	C*12:02	DRB1*14:06	DRB1*15:02	DQB1*03:01	DQB1*06:01	DPB1*05:01	DPB1*09:01
JPN40	A*24:02	A*24:20	B*07:02	B*13:01	C*03:04	C*07:02	DRB1*01:01	DRB1*14:07	DQB1*05:01	DQB1*05:03	DPB1*04:02	DPB1*05:01
JPN41	A*02:01	A*31:01	B*39:01	B*40:01	C*03:04	C*07:02	DRB1*04:03	DRB1*04:04	DQB1*03:02	DQB1*03:02	DPB1*02:01	DPB1*02:01
JPN42	A*24:02	A*26:01	B*40:01	B*40:02	C*03:04	C*03:23	DRB1*09:01	DRB1*09:01	DQB1*03:03	DQB1*03:03	DPB1*02:01	DPB1*05:01
JPN43	A*11:01	A*11:01	B*15:01	B*67:01	C*04:01	C*07:02	DRB1*04:06	DRB1*16:02	DQB1*03:02	DQB1*05:02	DPB1*02:02	DPB1*48:01
JPN44	A*02:06	A*24:02	B*27:05	B*52:01	C*01:02	C*12:02	DRB1*01:01	DRB1*15:02	DQB1*05:01	DQB1*06:01	DPB1*04:02	DPB1*09:01
JPN45	A*24:02	A*24:02	B*15:18	B*52:01	C*08:01	C*12:02	DRB1*13:07	DRB1*15:02	DQB1*03:01	DQB1*06:01	DPB1*03:01	DPB1*09:01
JPN46	A*02:01	A*24:02	B*07:02	B*35:01	C*07:02	C*08:01	DRB1*01:01	DRB1*11:19	DQB1*03:01	DQB1*05:01	DPB1*04:02	DPB1*04:02
