# Supplementary material for: Cost-efficient multiplex PCR for routine genotyping of up to nine classical HLA loci in a single analytical run of multiple samples by next generation sequencing
Source: BMC Genomics. 2015 Apr 18;16(1):318. doi: 10.1186/s12864-015-1514-4 (PMC4404632; doi:10.1186/s12864-015-1514-4)
Supplement: Additional file 5: Table S4. — Depth information of each allele and each locus. The short description of the data: Genotypes and average depth, average depth ratio, and depth per loci of 276 loci obtained by the 9LOCI method. [file 12864_2015_1514_MOESM5_ESM.pdf]

Table S4. Depth information of each allele and each locus

Locus	Sample ID	Allele1			Allele2			Average depth ratio	Depth per locus (obs)	Depth per locus (normalized)
		Allele name	Depth (obs)	Depth (nomalized)	Allele name	Depth (obs)	Depth (nomalized)			
HLA-A	JPN01	A*02:06:01	60.5	67.5	A*31:01:02	59.9	66.8	1.0	120.4	134.4
	JPN02	A*24:02:01	165.3	164.6	-	-	-	-	165.3	164.6
	JPN03	A*02:01:01	46.4	60.4	A*31:01:02	45.0	58.6	1.0	91.5	119.0
	JPN04	A*11:01:01	98.2	100.9	A*24:02:01	82.1	84.4	0.8	180.3	185.3
	JPN05	A*02:01:01	88.9	111.3	A*02:06:01	90.3	113.1	1.0	179.2	224.4
	JPN06	A*26:01:01	65.5	75.8	A*33:03:01	51.0	59.0	0.8	116.5	134.7
	JPN07	A*24:02:01	142.9	164.5	-	-	-	-	142.9	164.5
	JPN08	A*24:02:01	115.2	139.2	-	-	-	-	115.2	139.2
	JPN09	A*11:01:01	97.0	109.9	A*24:02:01	72.1	81.7	0.7	169.1	191.7
	JPN10	A*24:02:01	50.7	69.0	A*33:03:01	49.6	67.6	1.0	100.3	136.6
	JPN11	A*24:02:01	55.2	67.6	A*26:01:01	68.4	83.7	0.8	123.6	151.3
	JPN12	A*03:01:01	63.9	77.2	A*24:02:01	54.9	66.3	0.9	118.8	143.5
	JPN13	A*02:06:01	147.7	133.3	A*02:10:01	148.6	134.2	1.0	296.3	267.5
	JPN14	A*01:01:01	81.4	69.4	A*24:02:01	74.2	63.3	0.9	155.6	132.7
	JPN15	A*02:06:01	91.2	87.5	A*26:01:01	91.9	88.2	1.0	183.1	175.7
	JPN16	A*02:01:01	79.5	65.4	A*24:02:01	79.0	65.0	1.0	158.5	130.4
	JPN17	A*24:02:01	68.7	79.2	A*31:01:02	57.2	66.0	0.8	125.9	145.1
	JPN18	A*02:06:01	83.1	83.3	A*31:01:02	75.3	75.5	0.9	158.3	158.8
	JPN19	A*24:02:01	160.5	161.1	-	-	-	-	160.5	161.1
	JPN20	A*02:06:01	81.3	78.7	A*33:03:01	92.4	89.5	0.9	173.7	168.1
	JPN21	A*02:01:01	31.6	43.4	A*11:01:01	68.4	93.9	0.5	100.0	137.3
	JPN22	A*02:07:01	64.6	59.4	A*11:02:01	88.3	81.1	0.7	152.9	140.5
	JPN23	A*02:07:01	78.5	72.2	A*11:01:01	78.3	71.9	1.0	156.8	144.1
	JPN24	A*11:01:01	123.3	122.2	A*24:02:01	75.2	74.6	0.6	198.5	196.8
	JPN25	A*11:01:01	103.1	129.3	A*24:02:01	88.5	111.0	0.9	191.6	240.3
	JPN26	A*24:02:01	138.5	164.9	-	-	-	-	138.5	164.9
	JPN27	A*02:01:01	64.4	65.6	A*26:01:01	69.5	70.9	0.9	133.9	136.6
	JPN28	A*02:01:01	50.9	56.6	A*26:03:01	51.8	57.5	1.0	102.7	114.1
	JPN29	A*02:01:01	49.2	59.6	A*11:01:01	80.0	96.8	0.6	129.2	156.4
	JPN30	A*02:01:01	50.9	60.3	A*24:02:01	71.4	84.8	0.7	122.3	145.1
	JPN31	A*02:01:01	56.3	63.2	A*26:02:01	69.2	77.6	0.8	125.5	140.8
	JPN32	A*02:01:01	43.9	43.6	A*24:02:01	55.8	55.4	0.8	99.7	99.0
	JPN33	A*02:01:01	37.8	50.6	A*11:01:01	62.9	84.1	0.6	100.7	134.7
	JPN34	A*02:01:01	82.2	61.4	A*24:02:01	101.7	75.8	0.8	183.9	137.2
	JPN35	A*02:18	61.8	56.3	A*11:01:01	79.3	72.4	0.8	141.1	128.7
	JPN36	A*24:02:01	80.2	67.5	A*30:01:01	109.2	92.0	0.7	189.4	159.5
	JPN37	A*03:02:01	106.7	90.1	A*24:02:01	88.3	74.5	0.8	195.0	164.6
	JPN38	A*02:01:01	87.1	65.2	A*31:01:02	94.2	70.5	0.9	181.3	135.6
	JPN39	A*24:02:01	45.1	47.8	A*31:01:02	38.9	41.3	0.9	84.0	89.1
	JPN40	A*24:02:01	163.3	131.6	A*24:20:01	163.3	131.6	1.0	326.6	263.2
	JPN41	A*02:01:01	105.1	78.9	A*31:01:02	91.7	68.9	0.9	196.8	147.8
	JPN42	A*24:02:01	66.0	63.5	A*26:01:01	70.9	68.1	0.9	136.9	131.6
	JPN43	A*11:01:01	225.1	190.8	-	-	-	-	225.1	190.8
	JPN44	A*02:06:01	61.1	56.9	A*24:02:01	84.7	78.8	0.7	145.9	135.6
	JPN45	A*24:02:01	213.3	156.9	-	-	-	-	213.3	156.9
	JPN46	A*02:01:01	106.0	85.2	A*24:02:01	106.3	85.5	1.0	212.3	170.7
HLA-B	JPN01	B*39:01:01	38.0	42.5	B*40:02:01	70.4	78.6	0.5	108.5	121.1
	JPN02	B*07:02:01	141.2	140.6	B*55:02:01	108.4	108.0	0.8	249.7	248.6
	JPN03	B*40:06:01	67.6	88.0	B*48:01:01	105.2	137.0	0.6	172.8	224.9
	JPN04	B*15:01:01	122.0	125.3	B*55:02:01	87.5	89.9	0.7	209.5	215.2
	JPN05	B*35:01:01	102.2	127.9	B*51:02:01	108.7	136.1	0.9	210.8	264.0
	JPN06	B*40:02:01	80.0	92.5	B*44:03:01	109.3	126.4	0.7	189.3	218.8
	JPN07	B*07:02:01	113.7	130.9	B*15:07:01	84.2	96.9	0.7	197.9	227.9
	JPN08	B*13:01:01	92.2	111.4	B*40:03	69.8	84.3	0.8	162.0	195.7
	JPN09	B*35:01:01	129.7	147.0	B*56:01:01	120.2	136.2	0.9	249.9	283.3
	JPN10	B*07:02:01	90.4	123.0	B*58:01:01	59.4	80.8	0.7	149.7	203.8
	JPN11	B*48:01:01	130.1	159.3	B*54:01:01	57.1	69.9	0.4	187.2	229.2
	JPN12	B*07:02:01	117.6	142.0	B*44:02:01	89.8	108.5	0.8	207.4	250.6
	JPN13	B*15:01:01	133.4	120.4	B*40:06:01	96.3	87.0	0.7	229.7	207.4
	JPN14	B*37:01:01	54.6	46.6	B*51:01:01	132.1	112.7	0.4	186.7	159.2
	JPN15	B*35:01:01	114.1	109.5	B*40:02:01	108.9	104.5	1.0	223.0	214.0
	JPN16	B*15:18:01	199.6	164.3	B*35:01:01	147.8	121.7	0.7	347.5	286.0
	JPN17	B*51:01:01	156.8	180.8	B*52:01:01	153.9	177.4	1.0	310.7	358.2
	JPN18	B*39:01:03	51.7	51.9	B*40:02:01	79.7	79.9	0.6	131.4	131.8
	JPN19	B*07:02:01	154.0	154.6	B*39:04	49.6	126.4	0.3	203.6	281.0
	JPN20	B*58:01:01	107.9	104.5	B*59:01:01	81.3	78.7	0.8	189.3	183.2
	JPN21	B*35:01:01	75.1	103.1	B*56:03	88.3	121.2	0.9	163.4	224.3
	JPN22	B*27:04:01	98.1	90.2	B*46:01:01	114.7	105.4	0.9	212.8	195.5
	JPN23	B*46:01:01	341.8	314.2	-	-	-	-	341.8	314.2
	JPN24	B*35:01:01	117.3	116.2	B*39:01:01	51.7	51.3	0.4	169.0	167.5
	JPN25	B*15:01:01	96.9	121.6	B*38:02:01	49.1	61.6	0.5	146.0	183.1
	JPN26	B*15:01:01	192.2	228.9	B*15:27:01	193.9	193.9	1.0	386.1	422.8
	JPN27	B*40:01:02	155.3	158.4	B*54:01:01	69.4	70.8	0.4	224.7	229.2
	JPN28	B*15:01:01	161.1	178.9	B*46:01:01	154.8	172.0	1.0	315.9	350.9
	JPN29	B*35:01:01	121.0	146.4	B*40:02:01	67.7	82.0	0.6	188.7	228.4
	JPN30	B*52:01:01	105.5	125.2	B*55:04	90.5	107.4	0.9	196.0	232.6
	JPN31	B*35:01:01	81.6	91.6	B*40:01:02	113.9	127.8	0.7	195.5	219.4
	JPN32	B*35:01:01	94.2	93.5	B*39:02:01	33.7	33.5	0.4	127.9	127.0
	JPN33	B*15:01:01	140.3	187.7	B*15:11:01	143.6	192.2	1.0	283.9	379.8
	JPN34	B*15:01:01	210.7	157.2	B*40:50	168.7	125.9	0.8	379.5	283.1
	JPN35	B*15:01:01	234.2	213.5	B*46:01:01	233.0	212.4	1.0	467.2	426.0
	JPN36	B*13:02:01	150.5	126.7	B*51:01:01	140.8	118.6	0.9	291.3	245.2

	JPN37	B*13:02:01	179.2	151.2	B*40:06:01	139.2	117.5	0.8	318.4	268.7
	JPN38	B*40:02:01	128.4	96.0	B*51:01:01	164.5	123.1	0.8	292.9	219.1
	JPN39	B*39:23	49.7	52.7	B*52:01:01	131.8	139.8	0.4	181.4	192.5
	JPN40	B*07:02:01	168.3	135.6	B*13:01:01	148.7	119.8	0.9	317.0	255.5
	JPN41	B*39:01:03	46.9	35.2	B*40:01:02	166.5	125.1	0.3	213.5	160.4
	JPN42	B*40:01:02	138.3	132.9	B*40:02:01	93.2	89.6	0.7	231.5	222.5
	JPN43	B*15:01:01	138.5	117.5	B*67:01:01	46.1	39.1	0.3	184.6	156.5
	JPN44	B*27:05:02	88.3	82.1	B*52:01:01	115.4	107.3	0.8	203.7	189.4
	JPN45	B*15:18:01	153.2	112.7	B*52:01:01	133.8	98.4	0.9	286.9	211.1
	JPN46	B*07:02:01	165.4	133.0	B*35:01:01	118.2	95.0	0.7	283.6	228.1
HLA-C	JPN01	C*03:04:01	80.0	89.3	C*07:02:01	102.9	114.9	0.8	183.0	204.2
	JPN02	C*01:02:01	138.4	137.8	C*07:02:01	112.5	112.0	0.8	250.9	249.8
	JPN03	C*08:01:01	143.1	186.2	C*08:03:01	143.2	186.4	1.0	286.3	372.7
	JPN04	C*08:01:01	118.9	122.2	C*12:03:01	101.1	103.9	0.9	220.0	226.1
	JPN05	C*03:03:01	88.4	110.7	C*15:02:01	78.9	98.8	0.9	167.3	209.5
	JPN06	C*03:04:01	91.6	105.9	C*14:03	106.8	123.5	0.9	198.4	229.5
	JPN07	C*03:03:01	81.1	93.3	C*07:02:01	103.0	118.6	0.8	184.1	211.9
	JPN08	C*03:04:01	149.0	180.0	-	-	-	-	149.0	180.0
	JPN09	C*01:02:01	219.4	248.7	C*01:02:01	219.4	-	1.0	438.9	248.7
	JPN10	C*03:02:02	68.1	92.7	C*07:02:01	78.6	107.0	0.9	146.7	199.7
	JPN11	C*01:02:01	109.1	133.5	C*08:01:01	90.7	111.0	0.8	199.8	244.6
	JPN12	C*05:01:01	84.8	102.5	C*07:02:01	87.7	105.9	1.0	172.5	208.4
	JPN13	C*03:04:01	114.0	102.9	C*08:01:01	139.5	126.0	0.8	253.5	228.9
	JPN14	C*06:02:01	104.9	89.5	C*14:02:01	146.1	124.6	0.7	251.0	214.1
	JPN15	C*03:03:01	232.3	223.0	C*03:04:01	230.2	221.0	1.0	462.5	443.9
	JPN16	C*03:03:01	106.3	87.5	C*07:04-new	147.3	121.2	0.7	253.6	208.7
	JPN17	C*12:02:02	79.8	91.9	C*14:02:01	100.9	116.4	0.8	180.7	208.3
	JPN18	C*03:04:01	110.5	110.8	C*07:02:01	162.3	162.7	0.7	272.8	273.6
	JPN19	C*07:02:01	251.0	251.9	-	-	-	-	251.0	251.9
	JPN20	C*01:02:01	149.3	144.5	C*03:02:02	132.0	127.8	0.9	281.2	272.2
	JPN21	C*01:02:01	82.1	112.8	C*03:03:01	63.8	87.6	0.8	146.0	200.4
	JPN22	C*01:02:01	123.4	113.4	C*12:02:02	93.2	85.7	0.8	216.6	199.0
	JPN23	C*01:02:01	324.5	298.3	C*01:03	331.3	-	1.0	655.8	298.3
	JPN24	C*03:03:01	117.7	116.6	C*07:02:01	110.7	109.8	0.9	228.4	226.4
	JPN25	C*04:01:01	156.5	196.3	C*07:02:01	129.5	162.5	0.8	286.0	358.9
	JPN26	C*04:01:01	278.2	331.2	-	-	-	-	278.2	331.2
	JPN27	C*01:02:01	147.9	150.8	C*07:02:01	136.0	138.7	0.9	283.9	289.6
	JPN28	C*01:03	111.2	123.5	C*03:03:01	96.6	107.3	0.9	207.8	230.7
	JPN29	C*03:03:01	188.3	227.9	-	-	-	-	188.3	227.9
	JPN30	C*03:03:01	102.5	121.7	C*12:02:02	105.5	125.2	1.0	208.0	246.8
	JPN31	C*03:03:01	79.7	89.5	C*07:02:01	88.4	99.2	0.9	168.2	188.7
	JPN32	C*03:03:01	112.1	111.3	C*07:02:01	131.4	130.5	0.9	243.6	241.8
	JPN33	C*03:03:01	62.0	82.9	C*04:01:01	97.1	129.9	0.6	159.1	212.8
	JPN34	C*03:04:01	162.0	120.9	C*04:01:01	232.0	173.1	0.7	394.1	294.0
	JPN35	C*01:02:01	171.2	156.1	C*04:01:01	183.2	167.0	0.9	354.3	323.1
	JPN36	C*06:02:01	103.2	86.9	C*14:02:01	125.1	105.3	0.8	228.3	192.2
	JPN37	C*03:03:01	131.1	110.7	C*06:02:01	131.5	111.0	1.0	262.6	221.6
	JPN38	C*03:04:01	167.6	125.4	C*14:02:01	194.6	145.6	0.9	362.2	271.0
	JPN39	C*07:02:01	140.4	148.9	C*12:02:02	113.5	120.4	0.8	253.8	269.3
	JPN40	C*03:04:01	133.7	107.8	C*07:02:01	177.4	143.0	0.8	311.1	250.8
	JPN41	C*03:04:01	128.8	96.8	C*07:02:01	182.7	137.3	0.7	311.5	234.0
	JPN42	C*03:04:01	176.3	169.4	C*03:23:01	174.2	167.4	1.0	350.5	336.9
	JPN43	C*04:01:01	175.5	148.8	C*07:02:01	162.6	137.9	0.9	338.1	286.7
	JPN44	C*01:02:01	122.3	113.7	C*12:02:02	97.9	91.1	0.8	220.2	204.8
	JPN45	C*08:01:01	182.0	133.9	C*12:02:02	145.7	107.2	0.8	327.6	241.0
	JPN46	C*07:02:01	103.7	83.4	C*08:01:01	155.1	124.7	0.7	258.8	208.2
HLA-DRB1	JPN01	DRB1*08:03:02	270.9	302.4	DRB1*14:54:01	162.6	181.5	0.6	433.5	483.9
	JPN02	DRB1*01:01:01	348.7	347.2	DRB1*04:06:01	78.7	78.4	0.2	427.4	425.5
	JPN03	DRB1*09:01:02	326.7	425.2	-	-	-	-	326.7	425.2
	JPN04	DRB1*04:05:01	58.9	60.5	DRB1*09:01:02	182.0	187.0	0.3	240.9	247.5
	JPN05	DRB1*08:02:01	228.8	286.4	DRB1*11:01:01	154.8	193.9	0.7	383.6	480.3
	JPN06	DRB1*04:05:01	44.1	51.0	DRB1*13:02:01	192.3	222.4	0.2	236.4	273.3
	JPN07	DRB1*01:01:01	266.0	306.2	DRB1*04:06:01	94.6	108.9	0.4	360.6	415.1
	JPN08	DRB1*12:01:01	289.6	350.0	DRB1*12:02:01	300.1	362.6	1.0	589.7	712.6
	JPN09	DRB1*04:05:01	62.5	70.8	DRB1*11:01:01	194.6	220.5	0.3	257.0	291.3
	JPN10	DRB1*01:01:01	186.6	254.0	DRB1*13:02:01	177.9	242.2	1.0	364.5	496.2
	JPN11	DRB1*04:05:01	99.6	121.9	DRB1*04:07:01	102.0	124.8	1.0	201.6	246.7
	JPN12	DRB1*01:01:01	218.3	263.7	DRB1*13:01:01	234.9	283.8	0.9	453.2	547.5
	JPN13	DRB1*12:02:01	238.6	215.4	DRB1*15:01:01	211.1	190.6	0.9	449.7	406.0
	JPN14	DRB1*10:01:01	282.4	240.8	DRB1*14:03:01	280.3	239.0	1.0	562.6	479.8
	JPN15	DRB1*04:05:01	69.1	66.4	DRB1*11:01:01	271.3	260.4	0.3	340.5	326.8
	JPN16	DRB1*04:01:01	148.0	121.8	DRB1*04:10:03	149.0	122.7	1.0	297.1	244.5
	JPN17	DRB1*14:05:01	171.6	197.8	DRB1*15:02:01	150.7	173.8	0.9	322.3	371.6
	JPN18	DRB1*08:02:01	289.1	289.9	DRB1*12:02:01	261.8	262.6	0.9	550.8	552.5
	JPN19	DRB1*01:01:01	240.7	241.6	DRB1*09:01:02	219.6	220.4	0.9	460.3	462.0
	JPN20	DRB1*03:01:01	238.6	231.0	DRB1*04:05:01	48.7	47.1	0.2	287.3	278.1
	JPN21	DRB1*12:01:01	161.6	221.9	DRB1*15:01:01	110.0	151.0	0.7	271.6	372.9
	JPN22	DRB1*08:03:02	312.6	287.2	DRB1*12:01:01	288.9	265.4	0.9	601.5	552.6
	JPN23	DRB1*08:03:02	712.4	654.8	-	-	-	-	712.4	654.8
	JPN24	DRB1*08:03:02	331.7	328.8	DRB1*15:01:01	235.5	233.5	0.7	567.3	562.3
	JPN25	DRB1*04:05:01	65.0	81.5	DRB1*08:03:02	243.9	306.0	0.3	308.9	387.5
	JPN26	DRB1*04:06:01	77.0	91.7	DRB1*09:01:02	181.5	216.1	0.4	258.4	307.7
	JPN27	DRB1*04:05:01	82.8	84.5	DRB1*08:09	283.5	289.2	0.3	366.4	373.7
	JPN28	DRB1*09:01:02	349.6	388.2	-	-	-	-	349.6	388.2
	JPN29	DRB1*04:05:01	59.8	72.4	DRB1*15:01:01	175.5	212.4	0.3	235.3	284.8
	JPN30	DRB1*09:01:02	185.5	220.2	DRB1*15:02:01	155.3	184.3	0.8	340.8	404.4

	JPN31	DRB1*04:05:01	55.4	62.2	DRB1*12:01:01	237.4	266.3	0.2	292.8	328.5
	JPN32	DRB1*08:03:02	299.4	297.2	DRB1*09:01:02	240.9	239.2	0.8	540.3	536.4
	JPN33	DRB1*04:06:01	58.9	78.8	DRB1*09:01:02	207.5	277.6	0.3	266.4	356.4
	JPN34	DRB1*04:06:01	104.2	77.8	DRB1*08:02:01	367.0	273.8	0.3	471.3	351.6
	JPN35	DRB1*04:06:01	84.1	76.7	DRB1*08:03:02	314.7	287.0	0.3	398.8	363.6
	JPN36	DRB1*07:01:01	373.3	314.3	DRB1*14:03:01	281.0	236.6	0.8	654.3	550.9
	JPN37	DRB1*07:01:01	356.6	300.9	DRB1*12:01:01	275.6	232.6	0.8	632.1	533.5
	JPN38	DRB1*08:02:01	382.3	286.0	DRB1*14:02:01	337.8	252.7	0.9	720.1	538.7
	JPN39	DRB1*14:06:01	195.0	206.9	DRB1*15:02:01	197.0	209.0	1.0	392.0	415.9
	JPN40	DRB1*01:01:01	247.6	199.5	DRB1*14:07:01	269.7	217.3	0.9	517.3	416.9
	JPN41	DRB1*04:03:01	190.2	142.9	DRB1*04:04:01	191.6	143.9	1.0	381.8	286.8
	JPN42	DRB1*09:01:02	369.1	354.8	-	-	-	-	369.1	354.8
	JPN43	DRB1*04:06:01	86.8	73.6	DRB1*16:02:01	202.6	171.8	0.4	289.4	245.4
	JPN44	DRB1*01:01:01	293.8	273.2	DRB1*15:02:01	163.8	152.3	0.6	457.6	425.4
	JPN45	DRB1*13:07:01	360.0	264.8	DRB1*15:02:01	166.0	122.1	0.5	526.0	387.0
	JPN46	DRB1*01:01:01	368.6	296.4	DRB1*11:19:01	404.9	325.6	0.9	773.5	622.0
HLA-DQB1	JPN01	DQB1*05:02:01	182.3	203.5	DQB1*06:01:01	252.5	281.9	0.7	434.8	485.3
	JPN02	DQB1*03:02:01	202.9	202.0	DQB1*05:01:01	154.5	153.8	0.8	357.4	355.8
	JPN03	DQB1*03:03:02	293.9	382.5	-	-	-	-	293.9	382.5
	JPN04	DQB1*03:03:02	175.7	180.6	DQB1*04:01:01	134.5	138.2	0.8	310.2	318.8
	JPN05	DQB1*03:01:01	124.6	156.0	DQB1*04:02:01	87.9	110.0	0.7	212.5	266.0
	JPN06	DQB1*04:01:01	119.2	137.8	DQB1*06:04:01	180.9	209.2	0.7	300.1	347.0
	JPN07	DQB1*03:02:01	205.6	236.7	DQB1*05:01:01	155.8	179.4	0.8	361.4	416.1
	JPN08	DQB1*03:01:01	308.2	372.4	-	-	-	-	308.2	372.4
	JPN09	DQB1*03:01:01	145.9	165.4	DQB1*04:01:01	153.1	173.5	1.0	299.0	338.9
	JPN10	DQB1*05:01:01	105.5	143.6	DQB1*06:09:01	131.8	179.4	0.8	237.3	323.0
	JPN11	DQB1*03:02:01	186.5	228.3	DQB1*04:01:01	124.7	152.6	0.7	311.2	380.9
	JPN12	DQB1*05:01:01	149.4	180.4	DQB1*06:03:01	105.9	127.9	0.7	255.2	308.3
	JPN13	DQB1*03:01:01	221.1	199.6	DQB1*06:02:01	217.0	195.9	1.0	438.1	395.6
	JPN14	DQB1*03:01:01	150.2	128.1	DQB1*05:01:01	215.7	183.9	0.7	365.8	312.0
	JPN15	DQB1*03:01:01	156.1	149.8	DQB1*04:01:01	151.3	145.2	1.0	307.4	295.0
	JPN16	DQB1*03:01:01	191.3	157.5	DQB1*04:02:01	210.5	173.2	0.9	401.8	330.7
	JPN17	DQB1*05:03:01	150.2	173.2	DQB1*06:01:01	216.7	249.8	0.7	366.9	422.9
	JPN18	DQB1*03:01:01	164.1	164.5	DQB1*04:02:01	126.1	126.5	0.8	290.2	291.0
	JPN19	DQB1*03:03:02	176.1	176.8	DQB1*05:01:01	141.7	142.3	0.8	317.9	319.0
	JPN20	DQB1*02:01:01	146.2	141.5	DQB1*04:01:01	254.6	246.5	0.6	400.8	388.0
	JPN21	DQB1*03:01:01	85.1	116.9	DQB1*06:02:01	80.0	109.9	0.9	165.2	226.8
	JPN22	DQB1*03:01:01	146.0	134.1	DQB1*06:01:01	248.9	228.6	0.6	394.8	362.8
	JPN23	DQB1*06:01:01	565.7	519.9	-	-	-	-	565.7	519.9
	JPN24	DQB1*06:01:01	283.8	281.3	DQB1*06:02:01	190.9	189.2	0.7	474.6	470.5
	JPN25	DQB1*03:01:01	169.3	212.4	DQB1*04:01:01	157.0	197.0	0.9	326.3	409.3
	JPN26	DQB1*03:02:01	314.2	374.1	DQB1*03:03:02	307.2	365.8	1.0	621.3	739.8
	JPN27	DQB1*04:01:01	257.2	262.3	DQB1*04:02:01	259.6	264.8	1.0	516.8	527.1
	JPN28	DQB1*03:03:02	281.1	312.1	-	-	-	-	281.1	312.1
	JPN29	DQB1*04:01:01	84.8	102.7	DQB1*06:02:01	74.9	90.7	0.9	159.8	193.4
	JPN30	DQB1*03:03:02	157.9	187.3	DQB1*06:01:01	192.6	228.6	0.8	350.5	415.9
	JPN31	DQB1*03:01:01	141.4	158.6	DQB1*04:01:01	132.7	148.8	0.9	274.1	307.5
	JPN32	DQB1*03:03:02	208.9	207.4	DQB1*06:01:01	248.1	246.3	0.8	457.0	453.7
	JPN33	DQB1*03:02:01	239.6	320.6	DQB1*03:03:02	256.5	343.1	0.9	496.1	663.7
	JPN34	DQB1*03:02:01	614.4	458.4	-	-	-	-	614.4	458.4
	JPN35	DQB1*03:02:01	193.6	176.5	DQB1*06:01:01	255.0	232.5	0.8	448.6	409.0
	JPN36	DQB1*02:02:01	162.6	136.9	DQB1*03:01:01	287.2	241.8	0.6	449.8	378.7
	JPN37	DQB1*02:02:01	262.1	221.2	DQB1*03:01:01	362.4	305.8	0.7	624.4	527.0
	JPN38	DQB1*03:01:01	242.2	181.2	DQB1*04:02:01	238.0	178.1	1.0	480.2	359.3
	JPN39	DQB1*03:01:01	186.9	198.3	DQB1*06:01:01	223.4	237.0	0.8	410.3	435.3
	JPN40	DQB1*05:01:01	299.5	241.3	DQB1*05:03:01	313.0	252.3	1.0	612.4	493.6
	JPN41	DQB1*03:02:01	421.6	316.7	-	-	-	-	421.6	316.7
	JPN42	DQB1*03:03:02	340.8	327.5	-	-	-	-	340.8	327.5
	JPN43	DQB1*03:02:01	221.4	187.7	DQB1*05:02:01	210.2	178.2	0.9	431.5	365.9
	JPN44	DQB1*05:01:01	196.4	182.6	DQB1*06:01:01	285.8	265.7	0.7	482.2	448.3
	JPN45	DQB1*03:01:01	202.8	149.2	DQB1*06:01:01	355.2	261.3	0.6	558.0	410.5
	JPN46	DQB1*03:01:01	178.4	143.4	DQB1*05:01:01	202.3	162.7	0.9	380.6	306.1
HLA-DPB1	JPN01	DPB1*04:02:01	41.5	46.4	DPB1*05:01:01	45.6	50.9	0.9	85.2	97.3
	JPN02	DPB1*02:01:02	60.7	60.4	DPB1*13:01	93.5	93.1	0.6	150.7	153.5
	JPN03	DPB1*05:01:01	61.6	80.1	-	-	-	-	61.6	80.1
	JPN04	DPB1*02:02	106.7	109.6	-	-	-	-	106.7	109.6
	JPN05	DPB1*04:02:01	38.2	47.8	DPB1*05:01:01	35.4	44.3	0.9	71.9	92.1
	JPN06	DPB1*02:01:02	50.7	58.6	DPB1*04:01:01	53.2	61.5	1.0	101.5	120.1
	JPN07	DPB1*02:01:02	78.5	90.4	DPB1*04:02:01	84.5	97.3	0.9	159.4	187.7
	JPN08	DPB1*02:01:02	33.6	40.6	DPB1*05:01:01	45.3	54.7	0.7	77.1	95.3
	JPN09	DPB1*04:02:01	66.3	75.2	DPB1*05:01:01	72.8	82.5	0.9	136.0	157.6
	JPN10	DPB1*02:01:02	45.8	62.4	DPB1*05:01:01	59.4	80.8	0.8	102.9	143.2
	JPN11	DPB1*02:01:02	54.6	66.8	DPB1*19:01	45.6	55.8	0.8	97.9	122.6
	JPN12	DPB1*04:02:01	65.7	79.4	-	-	-	-	65.7	79.4
	JPN13	DPB1*05:01:01	106.9	96.5	-	-	-	-	106.9	96.5
	JPN14	DPB1*02:01:02	113.0	96.4	-	-	-	-	113.0	96.4
	JPN15	DPB1*05:01:01	65.5	62.8	DPB1*25:01	67.4	64.7	1.0	129.9	127.5
	JPN16	DPB1*02:01:02	65.6	54.0	DPB1*14:01	112.4	92.5	0.6	174.0	146.5
	JPN17	DPB1*09:01:01	110.0	126.8	DPB1*14:01	109.2	125.9	1.0	214.3	252.7
	JPN18	DPB1*02:01:02	48.3	48.4	DPB1*06:01	70.8	71.0	0.7	116.4	119.4
	JPN19	DPB1*04:02:01	66.5	66.7	DPB1*05:01:01	65.1	65.3	1.0	128.6	132.0
	JPN20	DPB1*04:02:01	61.1	59.2	DPB1*05:01:01	61.3	59.3	1.0	119.7	118.5
	JPN21	DPB1*02:01:02	28.8	39.5	DPB1*14:01	43.5	59.8	0.7	70.7	99.3
	JPN22	DPB1*05:01:01	98.6	90.6	-	-	-	-	98.6	90.6
	JPN23	DPB1*02:01:02	55.4	50.9	DPB1*05:01:01	61.0	56.1	0.9	113.8	107.0
	JPN24	DPB1*05:01:01	103.6	102.7	DPB1*38:01	103.1	102.2	1.0	202.1	204.9

JPN25	DPB1*05:01:01	50.7	63.6	DPB1*14:01	61.9	77.7	0.8	110.1	141.2
JPN26	DPB1*02:01:02	35.5	42.3	DPB1*14:01	64.1	76.3	0.6	97.4	118.6
JPN27	DPB1*05:01:01	116.3	118.6	-	-	-	-	116.3	118.6
JPN28	DPB1*05:01:01	66.6	73.9	-	-	-	-	66.6	73.9
JPN29	DPB1*02:01:02	34.3	41.6	DPB1*47:01	38.2	46.2	0.9	70.9	87.8
JPN30	DPB1*02:01:02	51.3	60.9	DPB1*05:01:01	70.3	83.4	0.7	118.9	144.3
JPN31	DPB1*02:01:02	36.9	41.3	DPB1*36:01	56.0	62.8	0.7	90.8	104.2
JPN32	DPB1*02:01:02	75.7	75.2	-	-	-	-	75.7	75.2
JPN33	DPB1*02:01:02	35.0	46.9	DPB1*05:01:01	38.0	50.9	0.9	71.4	97.7
JPN34	DPB1*02:01:02	46.5	34.7	DPB1*05:01:01	66.7	49.7	0.7	110.6	84.4
JPN35	DPB1*02:02	48.3	44.0	DPB1*05:01:01	46.4	42.3	1.0	92.6	86.3
JPN36	DPB1*17:01	76.0	64.0	DPB1*41:01:01	50.9	42.9	0.7	124.1	106.9
JPN37	DPB1*05:01:01	48.7	41.1	DPB1*17:01	42.2	35.6	0.9	88.9	76.7
JPN38	DPB1*02:02	71.5	53.5	DPB1*05:01:01	79.8	59.7	0.9	148.0	113.2
JPN39	DPB1*05:01:01	25.3	26.9	DPB1*09:01:01	38.4	40.7	0.7	62.3	67.6
JPN40	DPB1*04:02:01	50.2	40.5	DPB1*05:01:01	52.4	42.2	1.0	100.3	82.7
JPN41	DPB1*02:01:02	137.7	103.5	-	-	-	-	137.7	103.5
JPN42	DPB1*02:01:02	71.9	69.1	DPB1*05:01:01	72.2	69.4	1.0	140.8	138.4
JPN43	DPB1*02:02	67.4	57.2	DPB1*48:01	79.0	67.0	0.9	143.2	124.2
JPN44	DPB1*04:02:01	66.3	61.7	DPB1*09:01:01	73.7	68.5	0.9	136.9	130.2
JPN45	DPB1*03:01:01	144.0	105.9	DPB1*09:01:01	143.1	105.3	1.0	280.8	211.2
JPN46	DPB1*04:02:01	161.4	129.8	-	-	-	-	161.4	129.8
