# Supplementary material for: Cost-efficient multiplex PCR for routine genotyping of up to nine classical HLA loci in a single analytical run of multiple samples by next generation sequencing
Source: BMC Genomics. 2015 Apr 18;16(1):318. doi: 10.1186/s12864-015-1514-4 (PMC4404632; doi:10.1186/s12864-015-1514-4)
Supplement: Additional file 6: Table S5. — HLA allele frequency data in Japanese population. The short description of the data: A list of the HLA allele frequencies and ranking in the Japanese population. [file 12864_2015_1514_MOESM6_ESM.pdf]

Table S5. HLA allele frequency in Japanese population*

HLA-A n=21705 (48 alleles)

Allele	Freq.	Rank
*24:02	35.936%	1
*02:01	11.532%	2
*02:06	9.247%	3
*11:01	9.150%	4
*31:01	8.685%	5
*26:01	7.528%	6
*33:03	7.192%	7
*02:07	3.488%	8
*26:03	2.414%	9
*26:02	1.857%	10
*24:20	0.710%	11
*01:01	0.419%	12
*02:10	0.405%	13
*03:01	0.396%	14
*30:01	0.240%	15
*11:02	0.226%	16
*03:02	0.092%	17
*26:05	0.069%	18
*02:18	0.060%	19
*02:03	0.041%	20
*24:08	0.041%	20
*32:01	0.032%	22
*29:01	0.028%	23
*30:04	0.023%	24
*24:25	0.018%	25
*33:01	0.018%	25
*24:04	0.014%	27
*24:07	0.014%	27
*31:11	0.014%	27
*34:01	0.014%	27
*24:03	0.009%	31
*26:06	0.009%	31
*66:01	0.009%	31
*68:01	0.009%	31
*68:01	0.009%	35
*02:05	0.005%	36
*02:13	0.005%	36
*02:28	0.005%	36
*11:05	0.005%	36
*11:43	0.005%	36
*24:05	0.005%	36
*24:46	0.005%	36
*24:88	0.005%	36
*26:18	0.005%	36
*29:02	0.005%	36
*33:04	0.005%	36
*33:08	0.005%	36
*68:02	0.005%	36
*68:02	0.005%	49

HLA-B n=21705 (78 alleles)

Allele	Freq.	Rank
*52:01	11.080%	1
*51:01	9.039%	2
*35:01	8.233%	3
*40:02	7.828%	4
*15:01	7.782%	5
*54:01	7.593%	6
*44:03	6.492%	7
*07:02	5.612%	8
*40:01	5.266%	9
*46:01	4.842%	10
*40:06	4.639%	11
*39:01	3.377%	12
*48:01	2.843%	13
*55:02	2.543%	14
*59:01	2.013%	15
*15:18	1.484%	16
*13:01	1.249%	17
*67:01	1.212%	18
*15:11	0.963%	19
*56:01	0.931%	20
*58:01	0.599%	21
*15:07	0.590%	22
*37:01	0.553%	23
*40:03	0.424%	24
*44:02	0.396%	25
*13:02	0.327%	26
*38:02	0.290%	27
*39:02	0.286%	28
*27:04	0.221%	29
*56:03	0.203%	30
*39:04	0.198%	31
*51:02	0.198%	31
*55:04	0.129%	33
*15:27	0.088%	34
*27:05	0.069%	35
*15:02	0.041%	36
*07:05	0.037%	37
*38:01	0.028%	38
*14:02	0.023%	39
*14:01	0.018%	40
*39:23	0.018%	40
*08:01	0.014%	42
*15:25	0.014%	42
*15:38	0.014%	42
*35:05	0.014%	42
*40:50	0.014%	42
*50:01	0.014%	42
*15:03	0.009%	48
*15:28	0.009%	48
*15:35	0.009%	48
*35:64	0.009%	48
*07:31	0.005%	52
*15:04	0.005%	52
*15:08	0.005%	52
*15:13	0.005%	52
*15:26N	0.005%	52
*18:01	0.005%	52
*27:06	0.005%	52
*35:04	0.005%	52
*35:35	0.005%	52
*39:05	0.005%	52
*40:11	0.005%	52
*40:52	0.005%	52
*41:02	0.005%	52
*45:01	0.005%	52
*46:02	0.005%	52
*49:01	0.005%	52
*51:01V	0.005%	52
*51:03	0.005%	52
*51:06	0.005%	52
*51:36	0.005%	52
*52:11	0.005%	52
*53:01	0.005%	52
*55:10	0.005%	52
*55:12	0.005%	52
*57:01	0.005%	52
*78:02	0.005%	52
*82:02	0.005%	52

HLA-C n=12685 (28 alleles)

Allele	Freq.	Rank
*01:02	17.927%	1
*03:03	12.889%	2
*07:02	12.464%	3
*03:04	12.456%	4
*12:02	11.273%	5
*08:01	7.418%	6
*14:02	6.874%	7
*14:03	6.551%	8
*04:01	4.352%	9
*15:02	3.051%	10
*08:03	1.356%	11
*07:04	1.009%	12
*06:02	0.812%	13
*03:02	0.552%	14
*05:01	0.355%	15
*01:03	0.300%	16
*07:01	0.110%	17
*12:03	0.095%	18
*02:02	0.039%	19
*08:02	0.024%	20
*03:43	0.016%	21
*07:15	0.016%	21
*15:05	0.016%	21
*17:01	0.016%	21
*01:02V	0.008%	25
*04:15	0.008%	25
*15:10	0.008%	25
*16:04	0.008%	25

HLA-DRB1 n=21705 (43 alleles)

Allele	Freq.	Rank
*09:01	14.130%	1
*04:05	13.679%	2
*15:02	10.546%	3
*08:03	8.569%	4
*15:01	7.745%	5
*01:01	5.791%	6
*13:02	5.708%	7
*08:02	4.206%	8
*12:01	3.741%	9
*14:54	3.372%	10
*04:06	3.354%	11
*04:03	2.953%	12
*11:01	2.672%	13
*04:10	2.110%	14
*14:05	1.981%	15
*12:02	1.861%	16
*14:03	1.622%	17
*14:06	1.304%	18
*04:01	0.908%	19
*16:02	0.908%	19
*13:01	0.599%	21
*04:07	0.571%	22
*10:01	0.525%	23
*07:01	0.424%	24
*04:04	0.230%	25
*03:01	0.124%	26
*14:07	0.074%	27
*08:09	0.060%	28
*14:02	0.055%	29
*14:12	0.032%	30
*01:02	0.023%	31
*14:29	0.023%	31
*11:06	0.018%	33
*12:05	0.014%	34
*13:07	0.014%	34
*14:01	0.009%	36
*14:04	0.009%	36
*14:45	0.009%	36
*04:02	0.005%	39
*04:08	0.005%	39
*04:11	0.005%	39
*15:04	0.005%	39
*15:11	0.005%	39

HLA-DQB1 n=3016 (14 alleles)

Allele	Freq.	Rank
*06:01	19.032%	1
*03:03	15.451%	2
*04:01	12.832%	3
*03:01	11.538%	4
*03:02	9.483%	5
*06:02	7.228%	6
*05:01	6.631%	7
*06:04	5.206%	8
*04:02	4.344%	9
*05:03	3.979%	10
*05:02	2.586%	11
*06:03	0.597%	12
*02:01	0.564%	13
*06:09	0.531%	14

HLA-DPB1 n=2982 (19 alleles)

Allele	Freq.	Rank
*05:01	38.263%	1
*02:01	24.111%	2
*09:01	9.993%	3
*04:02	9.792%	4
*04:01	5.097%	5
*03:01	3.991%	6
*02:02	3.421%	7
*13:01	2.012%	8
*14:01	1.476%	9
*19:01	0.738%	10
*06:01	0.570%	11
*17:01	0.134%	12
*36:01	0.134%	12
*38:01	0.067%	14
*41:01	0.067%	14
*25:01	0.034%	16
*47:01	0.034%	16
*01:01	0.034%	16
*08:01	0.034%	16

*The data is released by HLA laboratory webpage (http://www.hla.or.jp/haplo/haplonavi.php?type=aril&lang=en).

**Gray background indicates HLA alleles included in the 46 DNA samples used in this study.
