# Supplementary material for: Cost-efficient multiplex PCR for routine genotyping of up to nine classical HLA loci in a single analytical run of multiple samples by next generation sequencing
Source: BMC Genomics. 2015 Apr 18;16(1):318. doi: 10.1186/s12864-015-1514-4 (PMC4404632; doi:10.1186/s12864-015-1514-4)
Supplement: Additional file 7: Table S6. — The number and frequency of HLA alleles in the Japanese population that were genotyped for the 46 Japanese reference DNA samples used in this study. The short description of the data: A list of HLA alleles frequencies in the Japanese population including the 46 DNA samples used in this study. [file 12864_2015_1514_MOESM7_ESM.pdf]

Table S6. The number and frequency of HLA alleles in the Japanese population that were genotyped for the 46 Japanese reference DNA samples used in this study.

HLA-A 18 alleles			HLA-B 37 alleles			HLA-C 18 alleles			HLA-DRB1 31 alleles			HLA-DQB1 14 alleles			HLA-DPB1 18 alleles		
Allele	Total Num.*	Freq.	Allele	Total Num.	Freq.	Allele	Total Num.	Freq.	Allele	Total Num.	Freq.	Allele	Total Num.	Freq.	Allele	Total Num.	Freq.
A*01:01	30	0.419%	B*07:02	488	5.612%	C*01:02	1059	17.927%	DRB1*01:01	503	5.791%	DQB1*02	16	0.564%	DPB1*02:01	1428	24.111%
A*02:01	695	11.532%	B*13:01	68	1.249%	C*01:03	16	0.300%	DRB1*03:01	5	0.124%	DQB1*03:01	583	11.538%	DPB1*02:02	229	3.421%
A*02:06	467	9.247%	B*13:02	11	0.327%	C*03:02	39	0.552%	DRB1*04:01	53	0.908%	DQB1*03:02	578	9.483%	DPB1*03:01	270	3.991%
A*02:07	213	3.488%	B*15:01	480	7.782%	C*03:03	829	12.889%	DRB1*04:03	184	2.953%	DQB1*03:03	972	15.451%	DPB1*04:01	281	5.097%
A*02:10	16	0.405%	B*15:07	36	0.590%	C*03:04	769	12.456%	DRB1*04:04	7	0.230%	DQB1*04:01	829	12.832%	DPB1*04:02	692	9.792%
A*02:18	2	0.060%	B*15:11	34	0.963%	C*03:23	3	**	DRB1*04:05	843	13.679%	DQB1*04:02	212	4.344%	DPB1*05:01	2350	38.263%
A*03:01	14	0.396%	B*15:18	65	1.484%	C*04:01	226	4.352%	DRB1*04:06	190	3.354%	DQB1*05:01	537	6.631%	DPB1*06:01	23	0.570%
A*03:02	1	0.092%	B*15:27	6	0.088%	C*05:01	17	0.355%	DRB1*04:07	25	0.571%	DQB1*05:02	131	2.586%	DPB1*09:01	700	9.993%
A*11:01	513	9.150%	B*27:04	5	0.221%	C*06:02	49	0.812%	DRB1*04:10	101	2.110%	DQB1*05:03	203	3.979%	DPB1*13:01	111	2.012%
A*11:02	4	0.226%	B*27:05	1	0.069%	C*07:02	873	12.464%	DRB1*07:01	11	0.424%	DQB1*06:01	1320	19.032%	DPB1*14:01	79	1.476%
A*24:02	2589	35.936%	B*35:01	482	8.233%	C*07:04	47	1.009%	DRB1*08:02	241	4.206%	DQB1*06:02	436	7.228%	DPB1*17:01	10	0.134%
A*24:20	24	0.710%	B*37:01	38	0.553%	C*08:01	407	7.418%	DRB1*08:03	516	8.569%	DQB1*06:03	23	0.597%	DPB1*19:01	28	0.738%
A*26:01	461	7.528%	B*38:02	12	0.290%	C*08:03	71	1.356%	DRB1*08:09	3	0.060%	DQB1*06:04	357	5.206%	DPB1*25:01	2	0.034%
A*26:02	97	1.857%	B*39:01	200	3.377%	C*12:02	871	11.273%	DRB1*09:01	928	14.130%	DQB1*06:09	33	0.531%	DPB1*36:01	2	0.134%
A*26:03	136	2.414%	B*39:02	10	0.286%	C*12:03	2	0.095%	DRB1*10:01	34	0.525%				DPB1*38:01	6	0.067%
A*30:01	10	0.240%	B*39:04	9	0.198%	C*14:02	425	6.874%	DRB1*11:01	118	2.672%				DPB1*41:01	12	0.067%
A*31:01	495	8.685%	B*39:23	1	0.018%	C*14:03	416	6.551%	DRB1*11:19	1	**				DPB1*47:01	6	0.034%
A*33:03	463	7.192%	B*40:01	311	5.266%	C*15:02	111	3.051%	DRB1*12:01	184	3.741%				DPB1*48:01	1	**
			B*40:02	529	7.828%				DRB1*12:02	89	1.861%						
			B*40:03	19	0.424%				DRB1*13:01	23	0.599%						
			B*40:06	274	4.639%				DRB1*13:02	389	5.708%						
			B*40:50	1	0.014%				DRB1*13:07	1	0.014%						
			B*44:02	17	0.396%				DRB1*14:02	1	0.005%						
			B*44:03	415	6.492%				DRB1*14:03	88	1.622%						
			B*46:01	290	4.842%				DRB1*14:05	112	1.981%						
			B*48:01	149	2.843%				DRB1*14:06	80	1.304%						
			B*51:01	508	9.039%				DRB1*14:07	4	0.074%						
			B*51:02	3	0.198%				DRB1*14:54	181	3.372%						
			B*52:01	865	11.080%				DRB1*15:01	454	7.745%						
			B*54:01	492	7.593%				DRB1*15:02	815	10.546%						
			B*55:02	120	2.543%				DRB1*16:02	46	0.908%						
			B*55:04	3	0.129%												
			B*56:01	33	0.931%												
			B*56:03	9	0.203%												
			B*58:01	39	0.599%												
			B*59:01	135	2.013%												
			B*67:01	72	1.212%												
	Total	99.576%		Total	99.622%		Total	99.732%		Total	99.788%		Total	100%		Total	99.933%

*Total Num. indicates allele numbers observed in the 6,230 HLA alleles per locus (the 3,115 donor samples).

**Gray background indicates HLA alleles lacked in the HLA frequency data in Japanese population shown in Table S5.
